# Prospects and challenges of deep learning in gynecologic malignancies

**DOI:** 10.3389/fonc.2025.1592078

**Published:** 2025-11-03

**Authors:** Yingfeng Zhang, Qin Qin

**Affiliations:** Obstetrics and Gynecology, University-Town Hospital of Chongqing Medical University, Chongqing, China

**Keywords:** artificial intelligence (AI), machine learning (ML), deep learning (DL), gynecological malignancies (GM), prospects and challenges

## Abstract

Artificial intelligence (AI) is revolutionizing oncology, with deep learning (DL) emerging as a pivotal technology for addressing gynecologic malignancies (GMs). DL-based models are now widely applied to assist in clinical diagnosis and prognosis prediction, demonstrating excellent performance in tasks such as tumor detection, segmentation, classification, and necrosis assessment for both primary and metastatic GMs. By leveraging radiological (e.g., X-ray, CT, MRI, and Single Photon Emission Computed Tomography (SPECT)) and pathological images, these approaches show significant potential for enhancing diagnostic accuracy and prognostic evaluation. This review provides a concise overview of deep learning techniques for medical image analysis and their current applications in GM diagnosis and outcome prediction. Furthermore, it discusses key challenges and future directions in the field. AI-based radiomics presents a non-invasive and cost-effective tool for gynecologic practice, and the integration of multi-omics data is recommended to further advance precision medicine in oncology.

## Highlights

- Synthesizes the integrated application of AI across multi-omics data (radiomics, pathomics, and genomics) for gynecologic malignancies, moving beyond siloed reviews.
- Details and contrasts a comprehensive array of both traditional machine learning and advanced deep learning architectures tailored for medical image and data analyses.
- Critically identifies the pervasive challenge of limited, heterogeneous data and the “black box” nature of AI as the primary barriers to clinical translation in GM care.
- Proposes standardized benchmarking and the development of explainable AI (XAI) frameworks as essential pathways for future clinical integration.
- Discusses the emerging role of graph neural networks (GNNs) in predicting drug synergism and analyzing complex biological networks for personalized therapy.

## 1 Introduction

Advances in computer science over the past decade have propelled the growth of artificial intelligence (AI), leading to its widespread adoption in various scientific domains, including medicine. AI differs from regular computer programming in several aspects. Traditional programming algorithms generate outputs based on input data and predefined rules, while AI has the ability to generate rules and patterns based on both input and output data. As a result, AI can accurately predict outcomes for fresh input.

**Figure 1:**
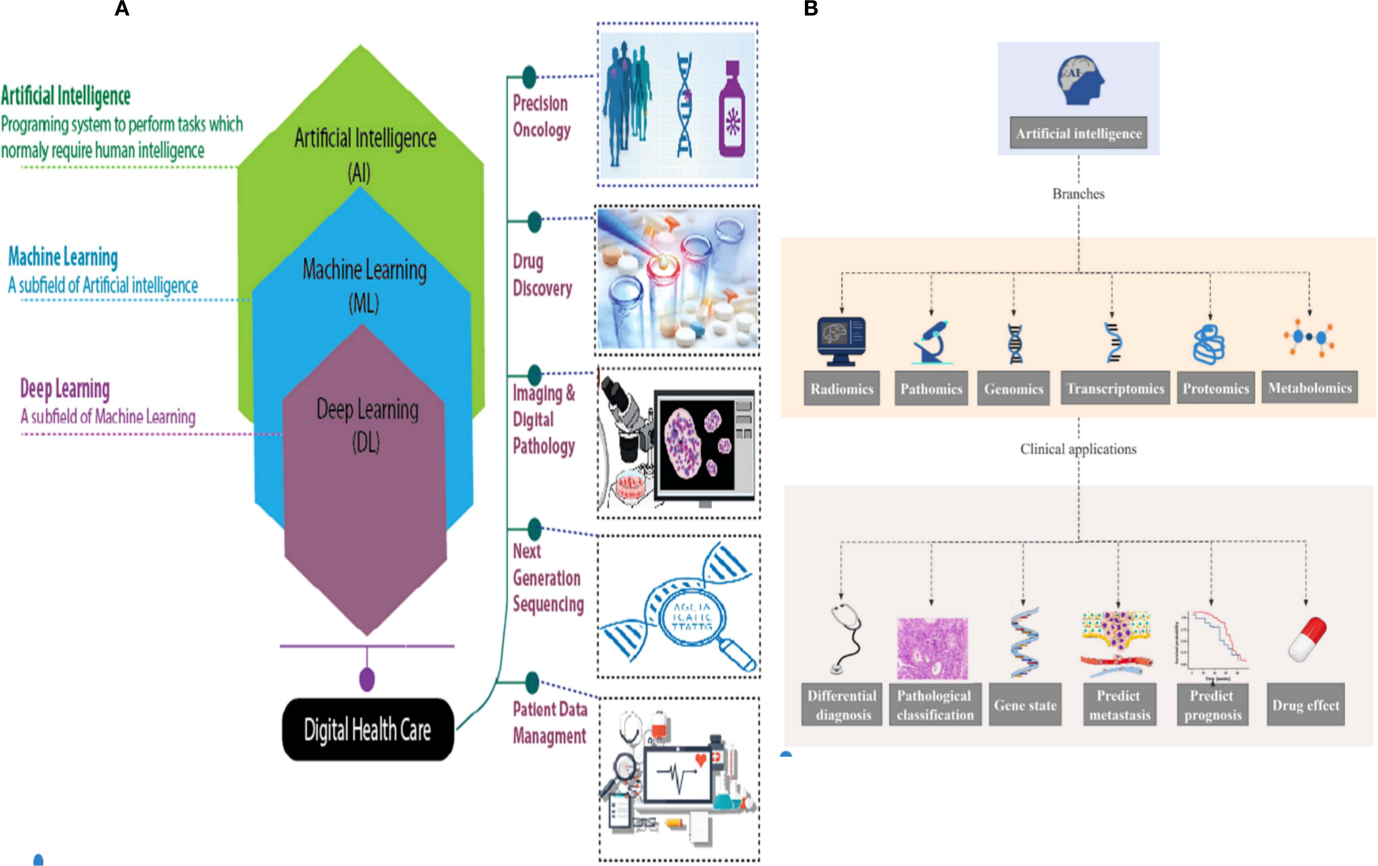
**(A)** Hierarchy of artificial intelligence (AI), machine learning (ML), deep learning (DL), and their applications in digital healthcare and oncology (e.g., precision oncology, drug discovery, and digital pathology) (3). **(B)** AI in omics (radiomics, pathomics, genomics, etc.) and related clinical applications (differential diagnosis, prognosis prediction, drug effect evaluation, etc.) (4).

AI and machine learning (ML) are increasingly making their presence felt in everyday life and are expected to have a significant impact on digital healthcare, particularly in the areas of disease detection and treatment, in the near future. The progress in AI and ML technologies has enabled the development of autonomous disease diagnosis tools. These tools utilize large datasets to address the future difficulties of the early identification of human diseases, particularly cancer. ML is a specific branch of AI that focuses on developing algorithms based on neural networks. These algorithms enable machines to learn and solve problems in a manner similar to the human brain (1). Deep learning (DL) is a subset of ML that aims to replicate the data processing capabilities of the human brain. It is used to detect images and objects, process languages, enhance drug discovery, improve precision medications, enhance diagnosis, and aid humans in decision-making. It is capable of functioning and generating outputs without human intervention (2). DL uses artificial neural networks (ANNs) to analyze data, such as medical images. It mimics the structure of the human neural system and consists of input, output, and hidden multi-layer networks. These networks improve the capabilities of machine learning processing (**Figure 1A**) (5).

**Figure 2:**
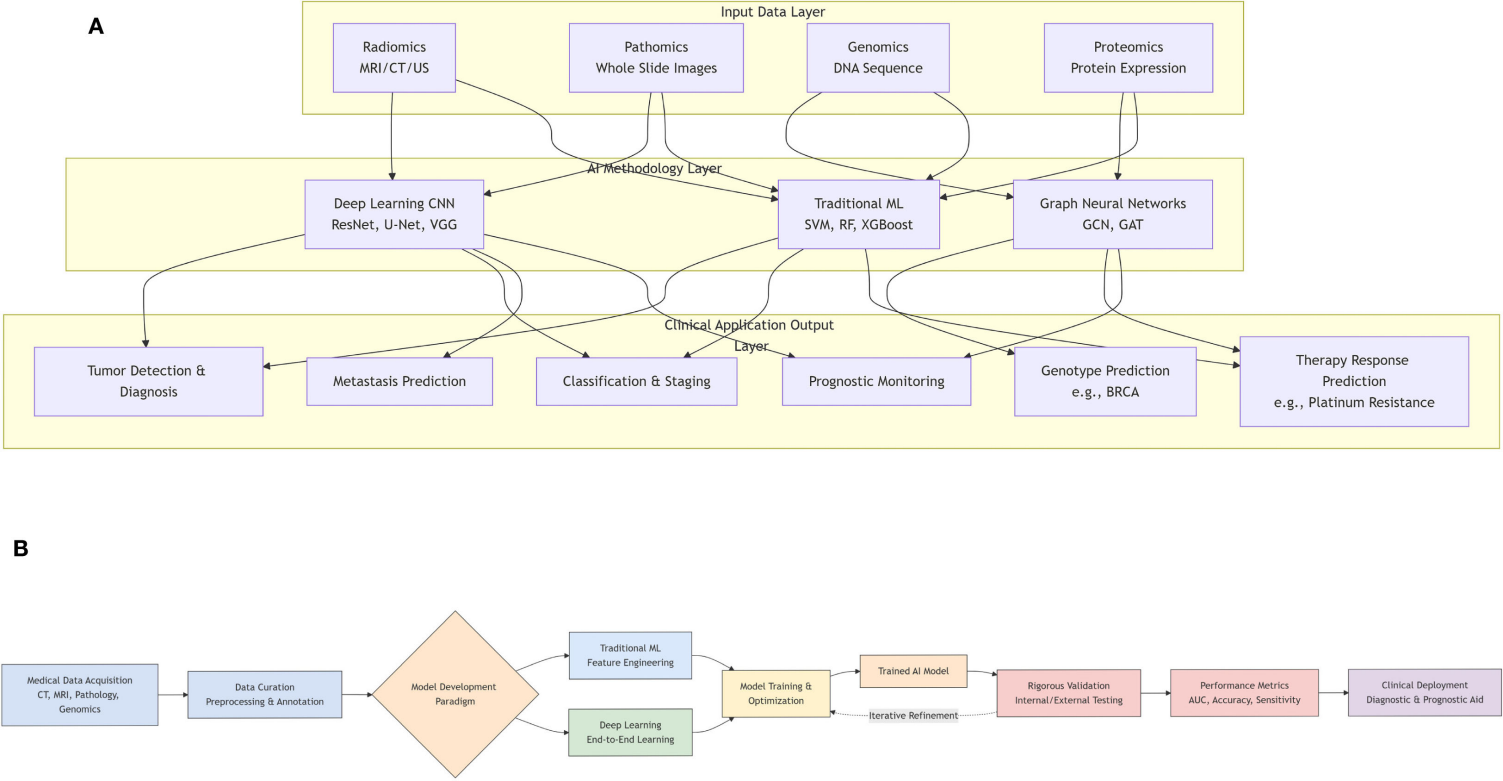
**(A)** Workflow of artificial intelligence (AI) model development and validation in medical research. **(B)** A graphical overview of the application of artificial intelligence in gynecologic malignancies.

The progress in artificial intelligence has led to the successful use of deep learning techniques, including segmentation, detection, classification, and augmentation, in the field of medical imaging (6, 7) (**Figure 2A**). This has opened up new possibilities for developing computer-aided systems for medical imaging diagnosis. Recent studies have shown that deep learning-based AI models can enhance the accuracy of diagnosing, predicting, and prognosticating gynecologic malignancies (GMs). These models also have the potential to improve the identification, classification, segmentation, and visual interpretation of bone tumors. In addition, radiomics is a sophisticated technology that is frequently used in conjunction with artificial intelligence. It is specifically developed to extract and analyze numerical radiological patterns using quantitative image parameters such as geometry, size, texture, and intensity. It is often compared to deep learning. Radiomics has been widely recognized as a valuable tool for disease prediction, prognosis, and monitoring (8).

When it comes to AI technology, gynecologic oncology falls short of the level required for everyday clinical use, unlike other medical specialties like endoscopy. The precise prediction of a definite diagnosis or prognosis significantly influences the therapy of gynecologic malignancies. The objective of this study was to elucidate the current status of AI research in relation to gynecologic cancers. In addition, we examined the obstacles encountered in the advancement of artificial intelligence in the field of gynecologic oncology. We anticipate that this study will subsequently encourage further research and accelerate the implementation of AI in the field of gynecologic oncology.

This review makes several key contributions to the field. First, it provides a comprehensive and up-to-date synthesis of the rapidly evolving application of deep learning across various imaging and omic modalities for gynecologic malignancies (**Figure 2B**). Second, it offers a detailed technical explanation of fundamental AI/ML/DL concepts and model architectures tailored for a clinical audience. Third, it critically examines not only the promising results but also the significant technical and clinical challenges hindering widespread clinical adoption. Finally, it discusses future directions to overcome these barriers and realize the potential of AI in improving gynecologic oncology care.

The paper is structured as follows: Section 2 introduces fundamental AI, ML, and DL concepts and architectures. Section 3 provides an overview of major gynecologic malignancies and precision oncology. Section 4 details DL applications in radiological image analysis (radiomics) for tasks like tumor detection, classification, and prognosis prediction. Section 5 focuses on DL for pathological image analysis, while Section 6 explores integration with other omics data. A comparative analysis of DL versus conventional imaging is presented in Section 7. Key technical and clinical challenges are discussed in Section 8, followed by future directions in Section 9. The review concludes with a summary in Section 10.

## 2 Artificial intelligence and deep learning

Artificial intelligence (AI) is a branch of computer science focused on replicating human intelligence to perform tasks that typically require human expertise (9). ML, a subset of AI, employs mathematical algorithms to enable autonomous decision-making (10). DL, a modern ML technique, differs from traditional ML in its data dependency, hardware requirements, feature engineering, problem-solving approach, execution time, and interpretability (11). DL excels in complex classification tasks using diverse inputs such as images, text, or audio, often outperforming classical ML methods (12). DL models consist of multiple layers that form neural network architectures and require extensive training on large labeled datasets.

### 2.1 Artificial intelligence

AI is an emerging discipline aimed at replicating, enhancing, and extending human intelligence through theoretical and technological innovations (13). The key components of AI technical systems include natural language processing, image recognition, human–computer interaction, and machine learning (14). Natural language processing integrates linguistics, computer science, and mathematics to enable machines to understand, interpret, and generate human language, supporting tasks such as information retrieval, speech recognition, and translation (15). Image processing involves acquisition, filtering, enhancement, and feature extraction, which significantly improves computational efficiency and reduces energy consumption compared to traditional methods (15). Human–computer interaction technologies, including computer graphics and augmented reality, facilitate seamless communication between users and machines (15). ML encompasses supervised, unsupervised, transfer, reinforcement, and integrated learning, employing algorithms such as deep learning, artificial neural networks, decision trees (16), and boosting algorithms. Rule-based AI systems have demonstrated clinical utility in lung cancer diagnosis (17), treatment (18), and prognosis (**Figure 1B**) (19).

AI is increasingly applied in medical research, including imaging, pathomics, genomics, transcriptomics, proteomics, and metabolomics. Recent studies have highlighted its role in multi-omics analysis for diagnosing GMs, distinguishing benign from malignant tumors, and predicting pathological classification, treatment response, and prognosis.

### 2.2 Machine learning

**Figure 3:**
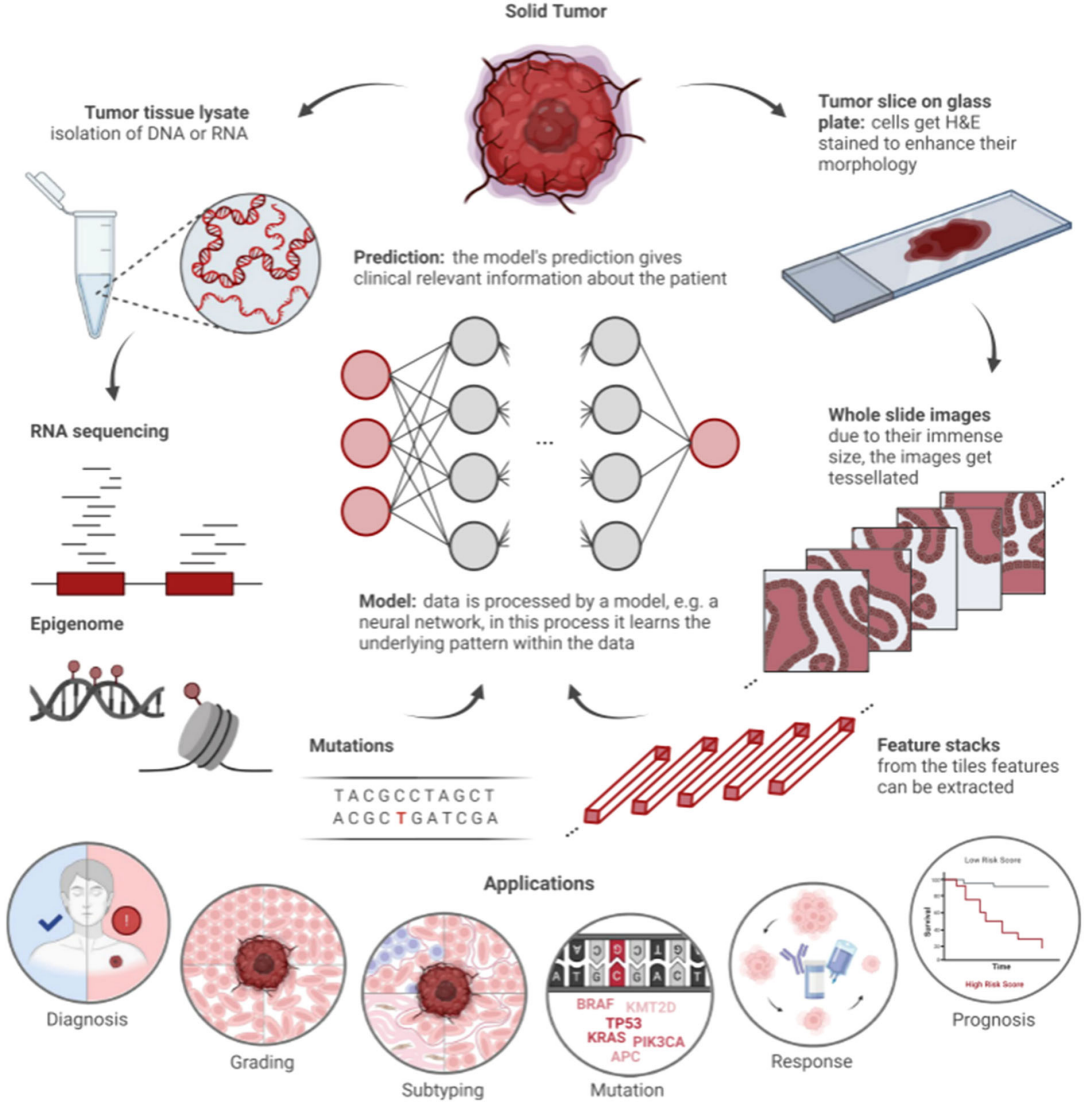
Workflow of artificial intelligence (AI) in histopathology and clinical genomics (21). Solid tumors supply two data streams: 1) molecular data (DNA/RNA sequencing, epigenomic profiles, and genetic mutations) and 2) histopathological data (H&E-stained tumor slices, with whole slide images split into tiles for feature extraction). A model (e.g., neural network) analyzes these data to identify patterns, supporting applications including diagnosis, grading, subtyping, mutation detection, treatment response prediction, and prognosis.

As a core component of AI, ML includes three primary methodologies: supervised, unsupervised, and reinforcement learning. In reinforcement learning, models receive rewards for correct decisions. Unsupervised learning identifies patterns in unlabeled data, such as through clustering algorithms. Supervised learning relies on human-labeled data to train models, which are penalized for incorrect predictions. Common supervised models include support vector machines (SVMs), decision trees, and artificial neural networks. These models vary in size, with neural networks containing parameters ranging from hundreds to billions (20). DL, or deep neural network, is particularly effective for image and text data due to its robustness in handling complex structures. In precision oncology, DL efficiently analyzes histopathologic and genomic data (**Figure 3**) (22). Multimodal approaches integrating ML and DL on diverse data types, such as histopathological images combined with genetic information, further enhance model performance by leveraging complementary information (**Table 1**) (42).

**Table 1:** Comparison of selected traditional machine learning and feature engineering methods.

Model/method	Key principle	Advantages	Limitations	Typical use cases in GMs
Support vector machine (SVM)	Finds the optimal hyperplane that maximizes the margin between classes.	Effective in high-dimensional spaces; robust against overfitting.	Sensitive to kernel/parameters; poor scalability to large datasets.	Classification of tumors based on radiomic features (23).
Random forest (RF)	An ensemble of decision trees, using bagging and feature randomness.	High accuracy; handles non-linear data; provides feature importance.	Less interpretable; computationally expensive with many trees.	Variable importance analysis; classification and regression (24, 25).
Decision tree (DT)	A tree-like model of decisions and their possible consequences.	Highly interpretable and visualizable; easy to understand.	Prone to overfitting; unstable to data variations.	Base learner for ensembles; preliminary data exploration (26, 27).
Artificial neural network (ANN)	Network of interconnected nodes that mimic neurons, learning complex non-linear relationships.	Can model highly complex patterns; universal function approximator.	Can be a black box; requires careful tuning; prone to overfitting without regularization.	Early ML models for classification and prediction tasks (28, 29).
k-Nearest Neighbor (k-NN)	Classifies a data point based on how its k-nearest neighbors are classified.	Simple to implement and understand; no training phase (lazy learner).	Computationally intensive during prediction; sensitive to irrelevant features and k-value.	Classification based on similarity in feature space (30, 31).
Bayesian network (BN)	A probabilistic graphical model representing variables and their conditional dependencies via a Directed Acyclic Graph (DAG).	Handles uncertainty well; interpretable causal relationships.	Learning network structure can be complex; requires prior knowledge or assumptions.	Probabilistic reasoning and risk assessment (32).
Classification and regression tree (CART)	A predictive model that uses a tree structure to go from observations to target value.	Can handle both classification and regression; handles non-linear relationships.	Can create over-complex trees that do not generalize well (overfitting).	Similar to DTs, used for building interpretable models (33).
Multivariate adaptive regression splines (MARS)	A non-parametric regression technique that models complex relationships by splitting data into regions.	Flexible in modeling non-linearities; handles high-dimensional data.	Can become overly complex and lose interpretability.	Modeling complex, non-linear relationships in medical data (34).
Gray-level co-occurrence matrix (GLCM)	A statistical method that examines texture by considering the spatial relationship of pixels.	Effective for capturing texture features in images; well-established.	Computationally heavy; features can be sensitive to image rotation and scale.	Texture analysis and feature extraction from MR/CT images (35).
Feature extraction	The process of transforming raw data into a reduced representation of informative features.	Reduces data dimensionality; can improve model performance and efficiency.	Hand-crafted features may not capture the most discriminative information.	Extracting radiomic features from medical images for downstream ML tasks (36–39).
Model building	The integrative process of combining features, clinical data, and algorithms to create a predictive model.	Creates robust and clinically applicable tools; can incorporate multi-modal data.	Requires domain expertise for variable selection and interpretation.	Building nomograms or integrated models for diagnosis/prognosis (40, 41).

GMs, gynecologic malignancies; ML, machine learning; DTs, decision trees.

#### 2.2.1 Support vector machine

SVM is a widely used ML method for classification and regression. It identifies the optimal hyperplane that separates classes in an n-dimensional space. The optimization objective for a linear SVM is as follows:


$$min┬\;(w,b)\;1/2|w{|}^{2},$$

$$s.t.\;y_{i}\;(w\cdot x_{i}+b)\geq 1,\;for\;all\;i=1,\;\ldots ,n,$$

where **w** is the weight vector defining the hyperplane, *b* is the bias term, **x**_i_ is the data points, and y_i_ ∈ {−1, +1} is their corresponding class labels.

SVMs use support vectors and kernel functions to handle non-linear separations. In GM research, SVMs have been applied to tumor detection using features from MR images (23).

#### 2.2.2 Decision tree

Decision trees (DTs) are supervised learning models that identify attributes and patterns in large datasets for predictive modeling (26). They provide interpretable visual representations of relationships between variables (27). While DTs are easy to construct and explain, ensemble methods like random forests improve predictive stability by combining multiple trees.

#### 2.2.3 Artificial neural network

ANNs are computational models inspired by biological neural networks, capable of learning patterns from data (28). They adapt through experience, making them suitable for classification and prediction tasks. ANNs exhibit non-linearity, enabling them to model complex data patterns. The output *a* of a neuron is computed as follows:


$$a=f(\sum_{\text{i}=1}^{n}w_{i}x_{i}+b),$$

where *x_i_* is the input, *w_i_* is the corresponding weight, *b* is the bias term, and *f* is the non-linear activation function (e.g., Sigmoid and Rectified Linear Unit (ReLU)). ANNs are structured into input, hidden, and output layers, with the configuration denoted as X–Y–Z, indicating the number of neurons in each layer (29).

#### 2.2.4 k-Nearest neighbor

k-Nearest neighbor (k-NN) is a non-parametric method used for classification and regression (30). It identifies the *k* most similar training examples to a new input and assigns the majority class among them. The choice of *k* affects model complexity: small *k* may lead to overfitting, while large *k* may include irrelevant data. Cross-validation helps select an optimal *k* (31).

#### 2.2.5 Bayesian network

Bayesian networks (BNs) represent probabilistic relationships among variables using a directed acyclic graph (32). Nodes denote variables, and arcs indicate dependencies. BNs estimate event probabilities rather than provide deterministic predictions.

#### 2.2.6 Random forest

Random forest (RF) is an ensemble learning method that combines multiple decision trees to reduce variance and improve accuracy (24). It trains trees on random data subsets and averages their predictions, mitigating overfitting and providing variable importance estimates (25).

#### 2.2.7 Classification and regression trees

Classification and regression tree (CART) constructs binary trees for classification or regression (33). Nodes represent decision rules, and leaves represent outcomes. Split points are chosen to minimize a cost function, emphasizing problem structure over data distribution.

#### 2.2.8 Multivariate adaptive regression splines

Multivariate adaptive regression splines (MARS) models relationships between continuous dependent and independent variables using piecewise regression equations (34). It handles categorical and continuous data, offering flexibility beyond linear regression.

#### 2.2.9 Gray-level co-occurrence matrix

Gray-level co-occurrence matrix (GLCM) is a texture analysis method that computes spatial relationships between pixel pairs in an image (35). It generates a co-occurrence matrix from which statistical features are extracted and applied in MRI-based feature analysis (35).

#### 2.2.10 Feature extraction

Feature extraction includes feature selection and transformation (36). Selection identifies relevant variables (e.g., gene expression), while transformation uses dimensionality reduction or neural networks to derive latent features (37–39). Graph neural networks [graph convolutional network (GCN), graph autoencoder (GAE), and graph attention network (GAT)] learn low-dimensional representations from network-structured data for predictive modeling.

#### 2.2.11 Model building

The final step in radiomics integrates clinical data, risk factors, biomarkers, and radiomic features into predictive models (e.g., nomograms) (40). Such models improve disease diagnosis, classification, and prognosis, advancing personalized medicine (41).

### 2.3 Deep learning

**Figure 4:**
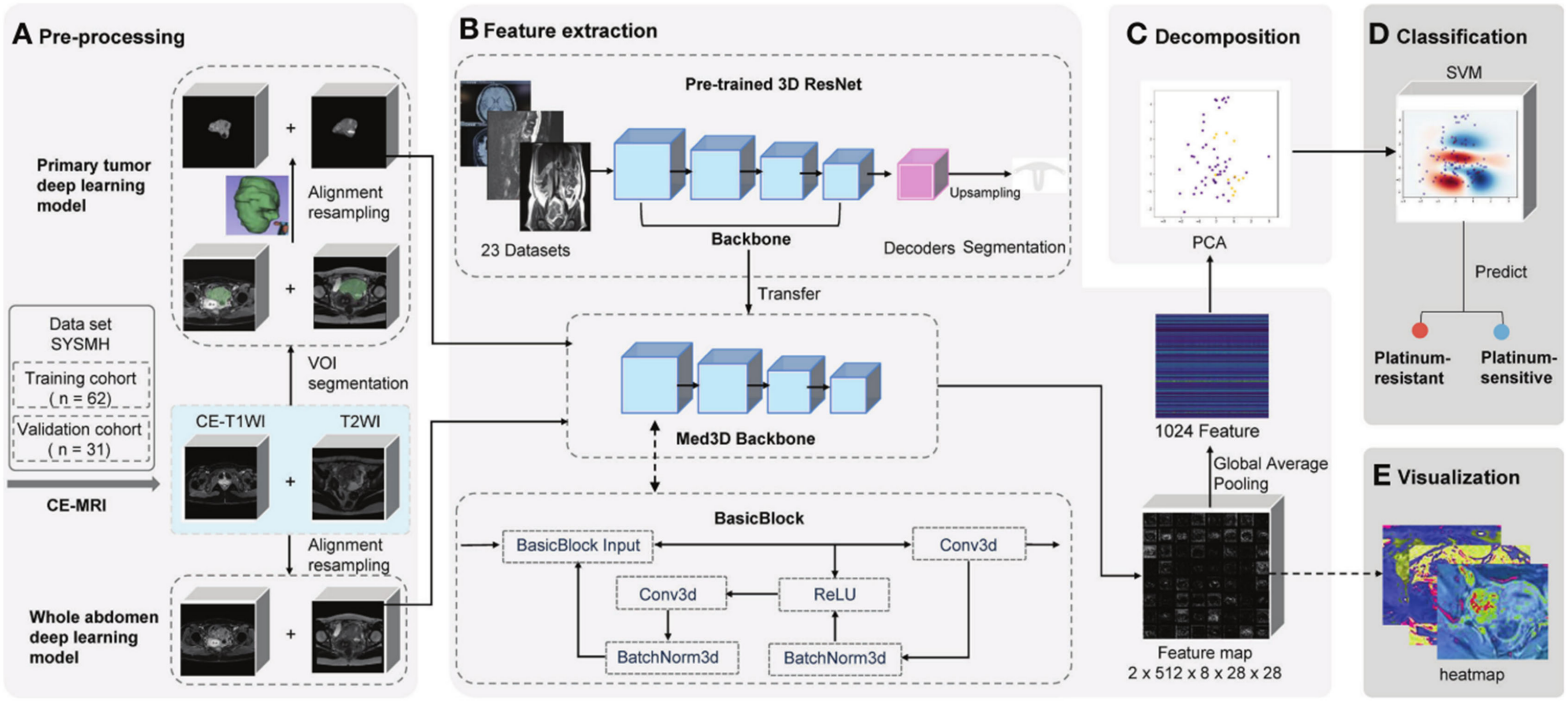
Schematic of deep learning frameworks for predicting platinum sensitivity using MRI (47). Two models use distinct volumes of interest (VOIs). 1) Primary tumor model: manually segmented primary tumor in contrast-enhanced T1-weighted imaging (CE-T1WI)/T2-weighted imaging (T2WI). 2) Whole abdomen model: entire abdomen volume (no manual segmentation). Workflow: **(A)** pre-processing (segmentation/registration/normalization of CE-T1WI/T2WI VOIs), **(B)** feature extraction via pre-trained 3D ResNet (transferred backbone + global average pooling to extract 1,024 features per patient), **(C)** principal component analysis (PCA) for feature decomposition, **(D)** support vector machine (SVM)-based platinum sensitivity prediction, and **(E)** heatmap visualization of convolutional layer feature maps.

DL is a powerful subset of machine learning that automatically learns hierarchical features from large-scale datasets, such as images, text, and audio. A typical DL model consists of an input layer, multiple hidden layers (e.g., convolutional, pooling, recurrent, and fully connected layers), and an output layer (43). The convolutional layers extract local patterns through learnable filters, pooling layers reduce spatial dimensions and enhance translational invariance, and fully connected layers integrate high-level features for final prediction (44–46). DL encompasses various advanced architectures, including deep neural networks (DNNs), autoencoders (AEs), deep belief networks (DBNs), convolutional neural networks (CNNs), recurrent neural networks (RNNs), and generative adversarial networks (GANs). Among these, CNNs have achieved remarkable success in visual recognition tasks and are increasingly applied in medical image analysis (**Figure 4**) (48).

#### 2.3.1 Convolutional neural networks

A CNN is a type of feedforward neural network commonly composed of convolutional layers, activation functions (e.g., ReLU), pooling layers, and fully connected layers (49). The convolutional filters operate on local receptive fields and share parameters across spatial locations, enabling efficient feature learning without manual design. This capability has proven highly effective in tasks such as tumor segmentation and classification in medical imaging (50). However, CNNs generally require large amounts of annotated data for training and are susceptible to overfitting. Regularization methods such as dropout, weight decay, and data augmentation are widely used to improve generalization (51).

##### 2.3.1.1 Basic structure

The fundamental building blocks of CNNs include the following.

Convolutional layers: These layers apply a set of learnable filters to the input. Each filter performs convolution operations across the input volume to produce feature maps highlighting specific patterns such as edges, textures, or complex shapes. The discrete convolution operation between an input image *I* (height *H* and width *W*) and a kernel *K* (size *kh*​×*kw*​) is defined as follows:


$$(I*K)(i,j=\sum_{m=0}^{k_{h^{-1}}}\sum_{n=0}^{k_{w^{-1}}}I(i+m,j+n)\cdot K(m,n).$$

This operation is performed across the entire image to produce a feature map, highlighting the locations where the kernel’s pattern is detected. This process allows the network to detect locally relevant patterns such as edges, textures, and shapes (52).

Pooling layers: Pooling (e.g., max pooling or average pooling) downsamples the feature maps, reducing computational burden and increasing receptive field size. It also contributes to model robustness against input variations (53).

Fully connected layers: After feature extraction and dimensionality reduction, the features are flattened and processed through one or more fully connected layers. These layers perform global reasoning and generate final outputs, such as class labels or regression values (54).

**Figure 5:**
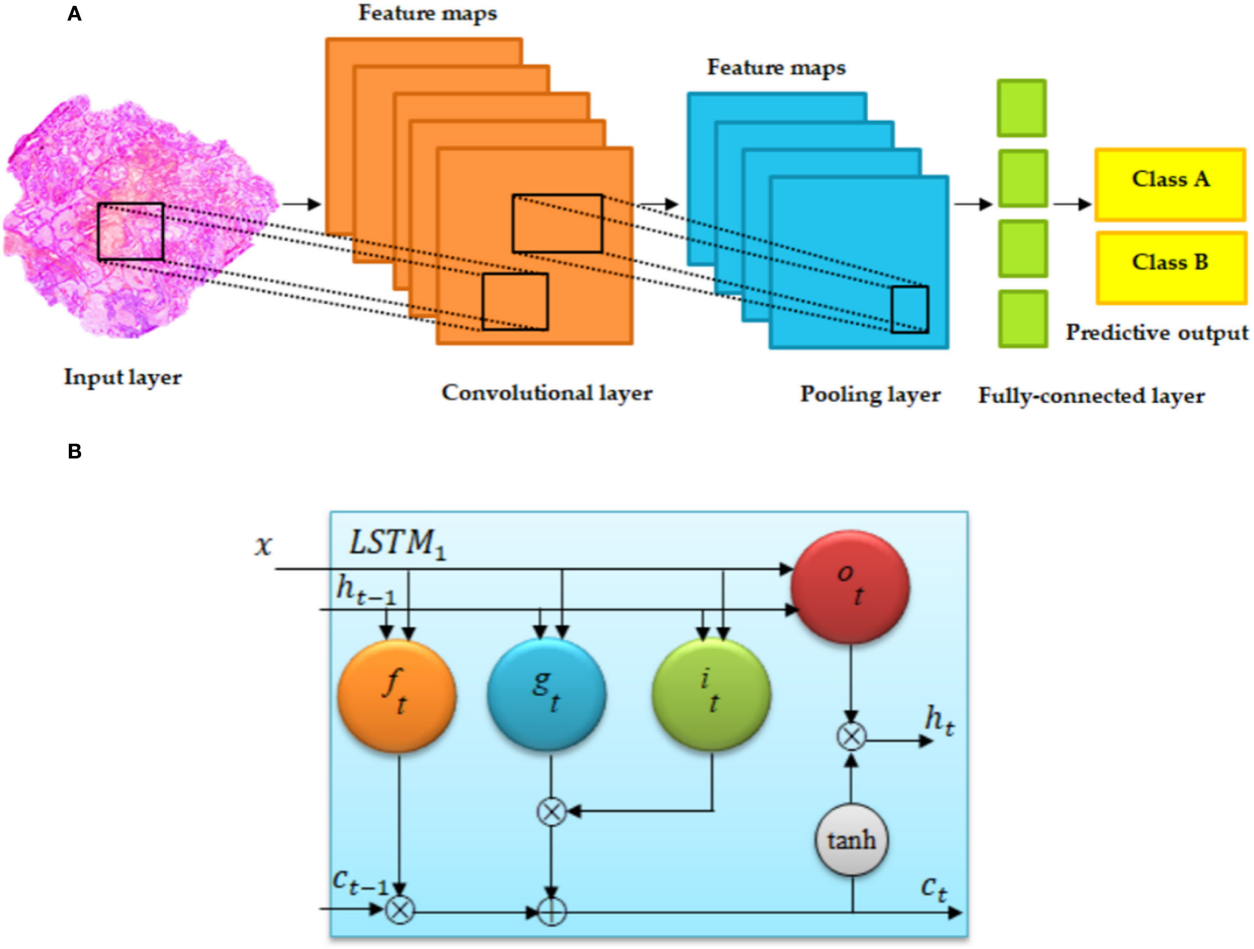
Basic architecture of **(A)** convolutional neural network and **(B)** long short-term memory (LSTM) (55). **(A)** Regular convolutional neural networks (CNNs) have convolutional, fully connected (FC), and pooling layers. **(B)** The memory cell c is controlled through a group of gate networks, including the following: f, forget gate network; i, input gate network; and o, output gate network.

A representative CNN structure is illustrated in **Figure 5**, showing the flow from input through convolutional and pooling layers to the fully connected output layers.

##### 2.3.1.2 Network structures

**Figure 6:**
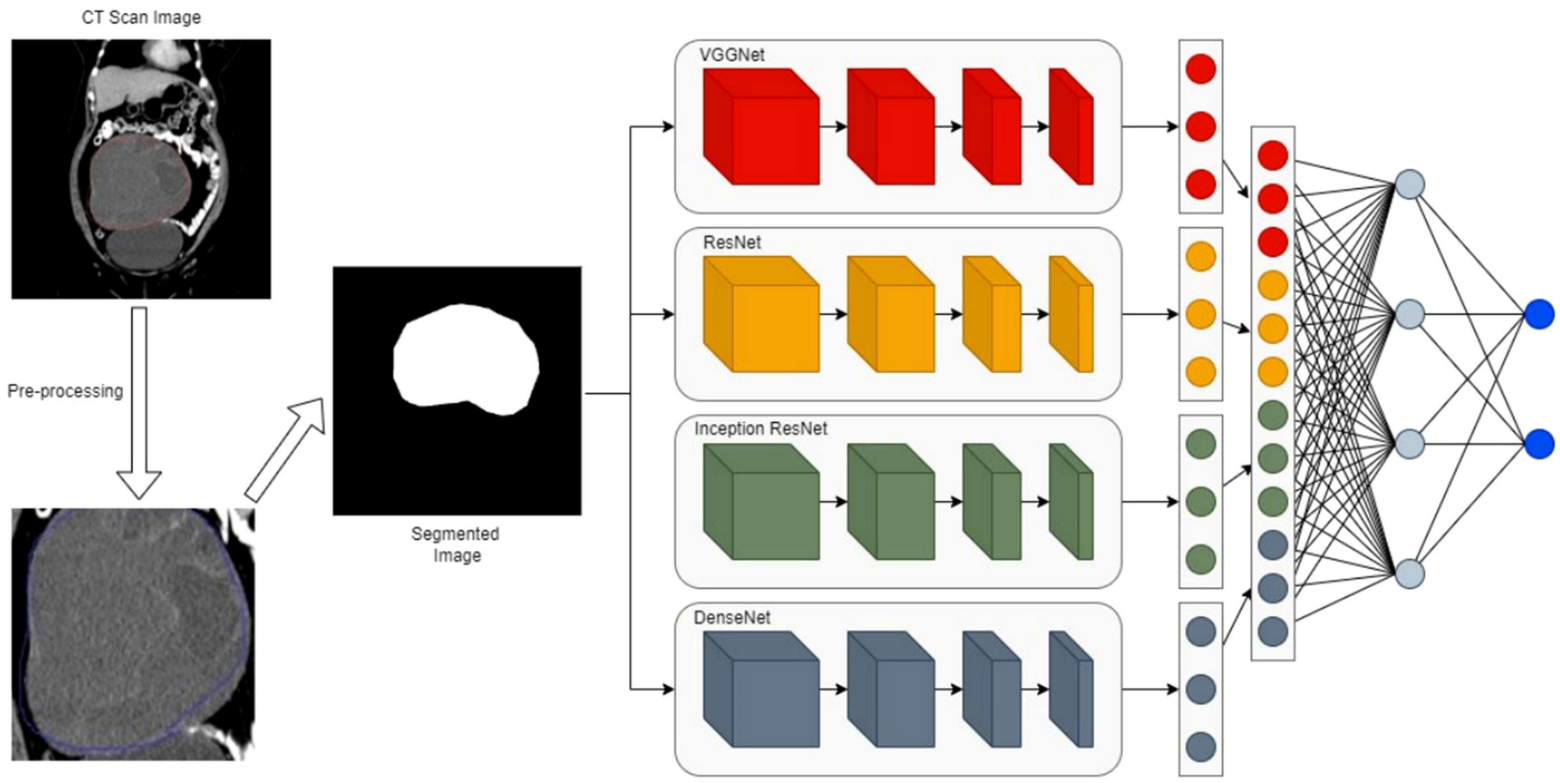
The proposed ensemble network with a four-path convolutional neural network (CNN) of VGGNet, ResNet, Inception, and DenseNet (74). The workflow involves 1) taking a CT scan image as input, 2) pre-processing the CT image to generate a segmented image of the region of interest, 3) extracting features from the segmented image via four parallel CNN branches, and 4) fusing branch-specific features and feeding them into subsequent networks to complete the final task (e.g., classification).

Convolutional, ReLU, pooling, and fully connected layers are stacked to form CNNs, which can be designed as either deep or shallow architectures. Classical deep CNNs such as LeNet (56), AlexNet (57), and GoogLeNet (58) are summarized in **Table 2**. Training deep CNNs requires large amounts of annotated data, which are often limited in medical imaging applications. Therefore, shallower CNN architectures are also widely considered in this domain, offering a balance between performance and data efficiency (**Figure 6**) (59).

**Table 2:** Comparison of selected deep learning model architectures.

Model	Key architecture	Advantages	Limitations	Typical use cases in medical imaging
AlexNet	A pioneering deep CNN with 5 convolutional and 3 fully connected layers, using ReLU activation.	Demonstrated the power of deep CNNs on large-scale datasets; revolutionized the field.	By modern standards, architecture is less efficient, and parameters are not optimized.	Baseline architecture for image classification tasks (57, 59).
VGGNet	A very deep CNN with a simple architecture using stacks of 3 × 3 convolutional layers.	High representational power due to depth; simple and uniform architecture.	Very computationally expensive and parameter-heavy due to full connections.	Feature extractor for various medical image analysis tasks.
GoogLeNet/Inception	Introduced the Inception module to perform multi-level feature extraction within a single layer.	Improved computational efficiency; reduced number of parameters.	Complex network design can be harder to modify and train from scratch.	Efficient and accurate image classification and detection (58).
ResNet	Introduces skip connections (residual blocks) to solve the vanishing gradient problem in very deep networks.	Enables training of extremely deep networks (100+ layers); state-of-the-art performance.	Very deep networks can still be computationally intensive.	Backbone for many state-of-the-art models in classification, segmentation, etc. (60).
U-Net	Symmetric encoder–decoder architecture with skip connections for precise localization.	Excellent for semantic segmentation; effective with limited data.	Primarily designed for segmentation, not classification.	Biomedical image segmentation (e.g., tumor and organ delineation) (61, 62).
Generative adversarial network (GAN)	Two networks (generator and discriminator) trained adversarially.	Can generate synthetic data; useful for data augmentation.	Training can be unstable (mode collapse).	Data augmentation for rare cancer types; image synthesis (63, 64).
Convolutional autoencoder (CAE)	An autoencoder using convolutional layers to encode input into a latent space and decode it.	Learns compressed representations; useful for denoising and dimensionality reduction.	The latent space may not be as interpretable.	Image denoising, compression, and unsupervised feature learning (65, 66).
Vision Transformer (ViT)	Applies transformer architecture with self-attention mechanisms to image patches.	Captures global contextual information effectively.	Requires large datasets to outperform CNNs; computationally heavy.	Alternative to CNNs for image classification and analysis (67).
Graph neural network (GNN)	A general class of networks that operate on graph-structured data.	Models complex relationships and dependencies between entities.	Not directly applicable to standard image data without graph construction.	Analyzing molecular structures, protein interactions, and relational data (68).
Graph convolutional network (GCN)	A type of GNN that performs convolution operations on graphs.	Efficiently captures node features and graph topology.	Requires a defined graph structure as input.	Node classification, link prediction in biological networks (69).
Graph attention network (GAT)	Incorporates attention mechanisms into graph learning, weighting the importance of neighbors.	Dynamic and adaptive neighborhood importance; often outperforms GCN.	Higher computational cost than GCN.	Tasks where some connections are more important than others (70).
Graph autoencoder (GAE)	Uses GNNs as encoders to learn node/graph embeddings for unsupervised reconstruction.	Learns meaningful latent representations of graph data in an unsupervised way.	Quality of embeddings is tied to the reconstruction task.	Dimensionality reduction, anomaly detection in network data (71).
GraphSAGE	An inductive framework that generates node embeddings by sampling and aggregating features from a node’s local neighborhood.	Generalizes to unseen nodes/graphs; not transductive like GCN.	Sampling process can omit important information.	Large-scale graph applications where new nodes are common (72).
Graph regularization	A technique to incorporate graph/network information as a constraint into an optimization problem.	Improves model performance by enforcing smoothness or structure on the solution.	Not a standalone model; an add-on technique to guide other algorithms.	Regularizing models in semi-supervised learning (73).

CNNs, convolutional neural networks.

It is essential to ensure that the test dataset follows the same distribution as the training set to obtain a reliable evaluation of model performance. Common metrics include accuracy, precision, recall, sensitivity, specificity, AUC-ROC, and F1-score. While accuracy is sometimes used for quick model comparison, a comprehensive evaluation typically employs multiple metrics. In practice, a trade-off between accuracy and computational efficiency (e.g., inference time within 100 ms) is often necessary, where accuracy is optimized under predefined runtime constraints (75).

**AlexNet:** This pioneering deep CNN helped popularize deep learning in computer vision. It consists of five convolutional layers and three fully connected layers, utilizing ReLU activations and dropout regularization. AlexNet significantly outperformed traditional methods in the ImageNet Large Scale Visual Recognition Challenge (ILSVRC) 2012 (59).

**VGGNet:** Known for its simplicity and depth, VGGNet uses stacks of 3 × 3 convolutional layers followed by max pooling. This design increases network depth while preserving receptive fields, improving feature learning capacity (31).

**GoogLeNet:** It introduced the Inception module, which performs parallel convolutions with different kernel sizes and merges their outputs. This structure captures multi-scale features efficiently while controlling computational cost (58).

**ResNet:** Residual Networks address the degradation problem in very deep networks through skip connections. These identity mappings allow gradients to flow directly through layers, enabling stable training of networks with hundreds of layers (60).

**U-Net**: Originally designed for biomedical image segmentation, U-Net employs a symmetric encoder–decoder architecture with skip connections. This design combines high-resolution features from the encoder with upsampled decoder features, enabling precise localization (61).

**GNNs and Extensions:** Graph neural networks (GNNs), including GCNs, GATs, GAEs, and GraphSAGE, extend convolutional operations to graph-structured data. They learn node representations by aggregating information from neighborhoods and have shown promise in modeling biological networks (68–72).

**GANs:** Generative adversarial networks consist of a generator and a discriminator trained adversarially. GANs are highly effective in generating realistic synthetic data and have been used for data augmentation, domain adaptation, and image reconstruction in medical applications (63, 64).

**CAEs:** Convolutional autoencoders employ convolutional layers in both encoder and decoder components. They are used for unsupervised representation learning, denoising, and anomaly detection (65, 66).

**ViT:** Vision Transformer (ViT)adapts the transformer architecture to images by dividing them into patches and processing them as sequences. ViT captures global contextual information and has achieved competitive performance in several medical imaging benchmarks (67).

## 3 Gynecologic malignancies

Timely identification can reduce the risk of substantial illness and death associated with neoplasms in women. Among all types of cancer, breast cancer is the most frequently occurring, with gynecologic malignancies of endometrial, ovarian, and cervical origin being the next most common (76). Although gynecologic cancers are less common than breast cancer, they have higher rates of illness and death. The American Cancer Society predicts that there will be approximately 116,760 new cases and 34,080 deaths caused by gynecologic cancers in 2021 (77).

The majority of existing studies using AI have concentrated on breast imaging. An extensive literature search on the application of AI in breast cancer imaging identified 767 studies spanning from the 1990s to the present. However, a different search for AI in gynecologic cancer imaging resulted in only 194 studies, with a majority of these being published in the last 2 years.

### 3.1 Ovarian cancer

Ovarian cancer (OC) typically manifests with a subtle onset, lacking any distinctive symptoms or indicators. Unfortunately, the disease often remains undetected until its advanced stages, affecting over 70% of patients. As a result, patients miss the window for effective treatment. As a result, ovarian cancer has the greatest death rate among tumors in the female reproductive system (78). The condition is characterized by mild initial symptoms and a poor prognosis. OC is the most prevalent and perilous form of gynecologic cancer. The four subtypes of primary epithelial ovarian carcinoma include serous, mucinous, endometrioid, and clear cell ovarian cancer. There is still a shortage of effective screening techniques for ovarian cancer. Clinical settings commonly employ the combination of transvaginal sonography and serum carbohydrate antigen (CA) 125 to initially identify ovarian cancer. However, this method has limited sensitivity and specificity (79). Transvaginal sonography frequently leads to the misidentification of benign pelvic masses as malignant ones (80), and its accuracy is significantly affected by the doctor’s level of expertise. However, peripheral blood testing offers the benefits of being painless, minimally invasive, and rapid, with greater acceptance and compliance. However, the use of CA125 is prone to false-positive results due to interference from benign tumors, inflammation, and hormone levels. Prior research (81) has consistently demonstrated that the area under the receiver operating characteristic curve (AUC) for the subject operating characteristic curve is below 0.8, posing challenges in meeting clinical requirements.

Debulking surgery and platinum-based chemotherapy are the conventional methods used to treat epithelial ovarian cancer (EOC) (82). Despite the possibility of achieving a high remission rate, approximately 20% to 30% of patients undergo numerous cycles of toxic medication before developing resistance to platinum-based treatments. This delay in identifying resistance and initiating therapy with effective drugs has proven to be a significant obstacle in improving patient outcomes (83). At the same time, platinum sensitivity is an easy way to find groups that respond to poly(ADP-ribose) polymerase inhibitors (PARPi) (84). This prediction can help prevent the unnecessary inclusion of patients in different clinical studies. If platinum sensitivity could be accurately anticipated, patients would derive greater advantages from precision therapy. Nevertheless, traditional clinical markers such as CA125 and tumor immunohistochemistry have a restricted ability to predict outcomes (85). In modern times, biopsies followed by mutation profiling or surgical resections have become a customary and enlightening practice (86). Nevertheless, the high expense, the invasive nature of the methods, the presence of genetic variation inside the tumor, and the need for many tumor samples greatly restrict the usefulness of molecular testing. This raises significant concerns about the cost-effectiveness of such testing.

The difficulty lies in the absence of a reliable screening technique, resulting in the diagnosis of ovarian cancer at an advanced stage, typically Stage III or IV. Radiologists conduct a manual analysis and interpretation of medical images from a patient suspected of having cancer in order to determine the specific type and stage of the cancer. As a result, the process misclassifies cancer subtypes, introduces variances in observations across different individuals, introduces subjectivity, and consumes a significant amount of time. This led to the creation of a diverse range of machine learning models aimed at forecasting and identifying tumors. The lack of effective early screening methods and the complexity of predicting platinum resistance represent significant clinical challenges that artificial intelligence and deep learning approaches are uniquely positioned to address.

### 3.2 Cervical cancer

The human cervix is lined by a delicate layer of tissue. The condition known as cervical cancer occurs when a cell transforms into a malignant one, exhibiting rapid growth and division, leading to the formation of a tumor. Early detection of this malignancy is crucial for successful treatment (87).

Cervical cancer is a prevalent type of cancer that affects the female reproductive system and has a significant impact on health and survival. It is widespread globally and particularly affects a large number of patients in China (88). Established risk factors for cervical cancer include human papillomavirus (HPV) infection, chlamydia infection, smoking, overweight/obesity, an unhealthy lifestyle, and the use of intrauterine devices (89). Prompt and consistent screening, together with early detection, are crucial in the prevention and management of cervical cancer. This is because precancerous abnormalities can manifest before the onset of cervical cancer and may progress into cancerous growths over a span of many years (90).

Cervical cancer screening involves the identification of cervical intraepithelial neoplasia (CIN), commonly referred to as cervical dysplasia. CIN is categorized into three grades: CIN1 (mild), CIN2 (moderate), and CIN3 (severe) (91). The main objective of cervical cancer screening in clinical practice is to assess the stage of CIN, which includes normal, CIN1, and CIN2/3.

Cervical cancer screening primarily consists of three steps: a Pap/HPV test, a colposcopy, and a pathological examination. During a Pap test, trained medical staff retrieve a few cell samples from the cervix and scrutinize them under a microscope to detect squamous and intraglandular epithelial lesions (SILs). The HPV test is a molecular test that pinpoints specific strains of the human papillomavirus associated with cervical cancer. If the Pap/HPV test yields abnormal results, it is recommended to undertake a colposcopy to locate suspicious lesions and undergo pathological investigations to determine the stage of CIN (92). Based on the specific attributes of the lesions seen during the colposcopy, the severity of CIN, and the patient’s medical background, a personalized treatment plan can then be developed.

Ultrasound is a commonly employed imaging diagnostic method for screening cervical cancer due to its simplicity and affordability. Computed tomography (CT) offers a superior ability to accurately display organs and soft tissue structures with subtle variations in density because of its high-density resolution. However, its capacity to evaluate the infiltration and dissemination of cervical cancer in other regions is limited, thereby constraining its therapeutic effectiveness. Because of the benefits of multiparameter multisequence imaging and high tissue resolution, MRI plain scan is highly suitable for diagnosing and staging cervical cancer (93). However, it still has certain drawbacks. Recently, researchers have developed several new multimodal MRI sequences that significantly enhance the diagnostic precision of MR images for various disorders.

Squamous cell carcinoma (SCC), adenocarcinoma (ADC), and tumors with unclear histological subtypes are the most common classifications for cervical cancer. SCC is the predominant form of cervical cancer, accounting for approximately 80% of all occurrences (94). SCC detection has greater clinical relevance for detecting SCC than ADC. HPV testing is more sensitive than cytology testing for cervical cancer screening, as discussed earlier (95). HPV testing enhances the comprehension of cervical cancer progression and identifies specific HPV genotypes, including HPV 16 and HPV 18. These two genotypes of high-risk HPV are the most prevalent, and together, they contribute to approximately 70% of cervical cancer cases. The Cancer Genome Atlas project has documented that gene alterations exhibit variability across different subtypes, indicating that distinct tumor subtypes may require tailored therapeutic interventions (96). The classification of cervical cancer subtypes is intriguing because it directly impacts the development of personalized treatment approaches by distinguishing between different types of cervical cells.

Although low-grade lesions often resolve on their own, high-grade lesions have the capacity to advance to aggressive malignancy. Hence, it is imperative to promptly detect high-grade lesions in order to intervene and prevent cervical cancer. DL algorithms can effectively and swiftly identify and categorize the extent of abnormalities in acetic acid test images, assisting in the prompt identification of severe abnormalities and enabling appropriate intervention and treatment. Computer-assisted diagnosis of cervical cancer is critical for efficiently preventing cancer development, making it highly important in clinical practice (97). However, the heavy reliance on cytological and colposcopic expertise, coupled with the subjective interpretation of screenings, creates a pressing need for automated, objective, and AI-powered diagnostic tools to improve accessibility and consistency in early detection.

### 3.3 Endometrial cancer

Endometrial carcinoma (EC) is a malignant tumor that develops in the inner epithelial lining of the uterus. It is the sixth most common cancer among women. Globally, 417,367 women received EC diagnoses in 2020, leading to significant financial burdens for both patients and caregivers (98). It is noteworthy that Asian women are prone to developing endometrial cancer at a younger age compared to other groups. Additionally, they tend to have more advanced stages of the illness. Therefore, it is crucial to accurately diagnose patients at an early age in order to provide appropriate management (99).

Endometrial cancer is associated with certain risk factors, such as postmenopausal hemorrhage, diabetes mellitus, arterial hypertension, smoking, nulliparity, and late menopause (100). Endometrial thickness has been proposed as a screening approach for women; however, its diagnostic effectiveness is severely limited due to the high occurrence of false-positive results, which have been reported to exceed 70% (101). Upon amalgamating several characteristics, the predictive efficacy of these indices appears to be enhanced, as indicated by recent studies reporting estimated sensitivity and specificity rates ranging from 70% to 80% in extensive cohorts (100).

The 2023 International Federation of Gynaecology and Obstetrics (FIGO) staging system categorizes endometrial cancers into two types: type I (low-grade endometrioid, grade 1 or 2) with a generally favorable prognosis and type II (grade 3 endometrioid, serous, clear cell, carcinosarcoma, undifferentiated/dedifferentiated) with a poorer prognosis. These tumors originate from many biological pathways with specific molecular changes (102). The current guidelines for optimum care of endometrial cancer patients include molecular categorization based on the standards published by the World Health Organization (WHO) (103), the European Society of Gynaecological Oncology (ESGO) (104), and the 2023 FIGO (105). WHO, the ESGO, and the 2023 FIGO guidelines all support the molecular categorization of endometrial cancer. This gives a more accurate prognosis and more personalized treatment plans than traditional grading methods. Nevertheless, the adoption of this technology is still limited, especially in underdeveloped nations, because of resource constraints and the limited availability of specialized diagnostic equipment. Molecular classification, in contrast to the grading system, focuses on examining precise genetic and molecular alterations (such as POLEmut, MMRd, NSMP, and p53abn) in cancer cells to inform treatment choices, rather than evaluating histological characteristics such as cellular atypia and tumor architecture.

Currently, the diagnosis of EC primarily relies on clinical symptoms, physical examinations, laboratory tests, transvaginal ultrasound, pelvic ultrasonography, endometrial biopsy with hysteroscopy, and various imaging techniques such as computed tomography, positron emission tomography/computed tomography, and magnetic resonance imaging. Diagnostic purposes also utilize certain biomarkers such as CA125 and HE4 (99). The goal of these examinations is to analyze the endometrial cells, assess the degree of disease, and identify the presence or absence of metastases. While these approaches exhibit favorable sensitivity in detecting EC, they also have drawbacks like limited specificity (especially transvaginal ultrasonography), invasiveness, discomfort, and high expense.

AI techniques in image processing are crucial for the timely identification, tracking, diagnosis, and treatment of EC. These methods aid the doctor in achieving a more precise disease diagnosis and can attain a high level of accuracy that may even surpass human recognition capabilities. Following the diagnosis of EC, physicians will endeavor to determine the extent of its dissemination, a procedure referred to as staging. The cancer stage refers to the extent to which cancer has spread across the body. It aids in assessing the severity of the cancer, devising appropriate treatment strategies, and predicting the potential efficacy of the treatment. The EC is categorized into four stages according to its extent of dissemination. The malignancy is localized exclusively in the uterus at stage 1. Stage 2 of cancer involves cancer cells in the uterus and cervix. In stage 3, the cancer has gone beyond the uterus but has not reached the rectum or bladder. It may also be found in the fallopian tubes, ovaries, vagina, and adjacent lymph nodes. The stage 4 malignancy has metastasized beyond the pelvic region. The presence of the condition may be observed in the bladder, rectum, and/or other remote tissues and organs. MRI is the most appropriate for detecting and assessing endometrial cavity EC, tumor infiltration into the myometrium, endocervix, and extensive spread into the parametria, as well as other cancer deposits in the pelvic region. Quantitative assessments on MRI are more effective than direct inspection by radiologists in identifying deep myometrial invasion. However, there are instances where it is not reliable to diagnose some invisible EC lesions on MRI. The rapid advancement of DL techniques, ranging from the initial shallow CNN model to the deep CNN model, along with the use of transfer learning, data augmentation, and other novel techniques, has provided motivation for their application in the automatic identification of EC.

Surgery remains a critical component in the treatment of endometrial cancer. The primary goals of this procedure are twofold: first, to remove the original tumor, and second, to accurately determine the extent of the disease and assess its prognostic aspects. While achieving the first target may be possible by a “simple” hysterectomy, the latter requires a more thorough intervention. This includes a complete omentectomy, pelvic lymphadenectomy, and lumbo-aortic lymphadenectomy (106). However, the therapeutic value of these procedures is still a subject of controversy. Performing invasive surgery on obese, elderly, and fragile individuals with endometrial cancer may result in serious consequences of significant concern. Therefore, it is necessary to maximize the diagnostic performance before surgery. Improving the patient selection process for surgery would lead to a decrease in the risk of unnecessary treatment, complications, and death by providing personalized care. The critical challenges of pre-operative molecular classification and accurate staging for personalized treatment planning are areas where deep learning models applied to imaging and histopathological data show immense potential.

### 3.4 Precision oncology

Precision tumor medicine entails utilizing a variety of advanced detection technologies, such as proteomics, transcriptomics, genomics, epigenomics, and metabonomics, to gather biological information related to tumors. This information is then used to guide the process of tumor screening, diagnosis, and treatment (107). The discovery of many gene mutations has significant benefits for molecular subtype classification, risk prediction for GMs, and accurate treatment strategy selection.

Precision oncology refers to the accurate identification and analysis of individual tumor cells. It is widely recognized as a crucial therapeutic approach in the battle against cancer and is centered on the identification of precise molecular targets. Precision oncology is associated with the use of personalized cancer genetic data. Additionally, it can incorporate proteomics data by extracting clinical signatures from electronic records stored in various computational databases (108). AI-based, innovative molecular techniques have been utilized in recent advancements in clinical oncology. Next-generation sequencing (NGS) is the optimal platform for producing large-scale datasets with high throughput. In addition, the development of an algorithm for early-stage cancer detection necessitates the involvement of oncology experts who possess a background in ML. This algorithm aims to identify new biomarkers and target sites, enable accurate diagnosis through NGS, identify specific target sites, and enhance medical imaging technology with high resolution (109). Precision oncology medications are developed to selectively attack cancer cells by exploiting their genetic heterogeneity. The system may use NGS data to recommend personalized therapy by taking into account individual genetic characteristics. AI is considered one of the leading cutting-edge treatments for accurate cancer diagnosis, prognosis, and treatment. This is achieved by analyzing large datasets from pharmaceutical and clinical sources through systematic data processing. The future of digital healthcare and clinical practices is expected to shift toward the utilization of algorithm-based AI for radiological image interpretation, e-health records, and data mining. This transformation aims to provide more accurate solutions for cancer treatment. The integration and interpretation of complex, high-dimensional multi-omics data remain a major hurdle in realizing the full promise of precision oncology, a challenge that requires the sophisticated pattern recognition capabilities of advanced AI and deep learning algorithms.

## 4 Deep radiomics-based learning in gynecologic malignancies

Radiomics is a method that allows for the extraction of a large number of imaging characteristics from medical images obtained by non-invasive procedures such as CT, MRI, and ultrasound. This methodology was initially introduced by Lambin et al. in 2012 (110). Medical images store large amounts of digital data that pertain to the pathophysiology of tumors (111). Furthermore, radiomics can extract pertinent characteristics from images and integrate and enhance the findings with clinical, pathophysiological, and molecular biological information. This can lead to enhanced clinical diagnosis, the prediction of tumor stage and genotype, and the assessment of prognosis (112). The primary stages of radiomics encompass medical image collecting, image segmentation, feature extraction, feature screening, and model development. Radiomics has been extensively employed in the investigation of many types of tumors, such as thyroid, breast, liver, prostate cancer, and OC (4).

The radiological evaluation could be enhanced by employing radiomics to characterize tumors. These imaging features are both reproducible and quantitative, and they enable the non-invasive evaluation of the heterogeneity of the tumor (113). AI in radiology is a newly developed field that involves the efficient extraction of digital medical imaging data to gather predictive and/or prognostic information about patients and their diseases. This is achieved by analyzing tumor heterogeneity and indirectly assessing the molecular and genetic features of the tumor. It has the potential to enable the anticipation of diagnosis, treatment response, and prognosis. The topic of research in oncology is rapidly growing in popularity due to its broad and potential applications, particularly in clinical decision-making and personalized treatment (114). A robust association exists between radiomic data and clinical results. The efficacy of this notion has already been demonstrated in predicting several solid tumors prior to surgery (115).

The AI methodology diverges from the usual radiological method by providing an automated, replicable, and quantitative examination of images that surpasses human visual capabilities. AI systems can be trained to analyze predefined criteria, such as tumor size, tumor shape, and lymph nodes, using machine learning. Alternatively, they can be educated without human supervision using DL, which involves a flexible analytical process that may not be easily understandable by humans. An instance of a free analysis chain is the artificial neural network, which has interconnected functions that process images as input and generate analysis as output. The complexity of a neural network may vary depending on the purpose and the type of input. A neural network is referred to as “deep” when it consists of multiple layers, known as “hidden layers”, through which information is transmitted. The greater the number of hidden layers in a network, the deeper and more intricate it becomes. An excessively complex model has the capability to fit extremely well to a particular training dataset, but it runs the risk of performing poorly when presented with fresh information. This phenomenon is sometimes referred to as “overfitting”. Hence, several methods of internal and external validation are employed to mitigate this issue, which compromises the algorithm’s applicability. The term “DL” pertains to the utilization of deep neural networks.

### 4.1 Tumor lesion detection and diagnosis

Initial tumors exhibit no distinct symptoms. Various forms of tumors may be accompanied by certain symptoms. Early detection of symptoms allows for the possibility of early detection of malignant tumor growth. When there is a suspicion of a tumor, a thorough examination can be conducted to obtain a comprehensive and unbiased diagnosis of the tumor’s state, facilitate early treatment, and enhance the chances of a cure.

Computer-aided diagnosis in the medical profession enables clinicians to convert subjective image data into objective image data, facilitating clinical decision-making. Nevertheless, DL utilizing a CNN possesses evident benefits in comparison to conventional computer-aided diagnosis. Simplifying the extraction procedure allows for the automatic extraction of distinctive feature information from datasets. Additionally, its performance is more systematic and offers greater ease of adjustment. ML and deep data mining techniques facilitate the identification of cancer by enabling researchers to extract distinctive information from the data, which may then be used for cancer prediction (31). The application of AI extends beyond detection to nuanced prognostic prediction. Studies have demonstrated that deep learning models can decode the complex interplay within the tumor microenvironment, offering insights into disease aggression and patient outcomes that surpass traditional staging systems (116). This aligns with our findings on the prognostic value of AI-derived features.

DL primarily utilizes X-ray, CT, and MR images for lesion detection and classification. Plain radiographs, generated using X-ray technology, provide image metrics that describe tumor features such as tumor location, tumor size, and tumor margin. CT and MRI offer enhanced radiological information and enhance the ability to detect lesions, in comparison to simple radiographs. Several advanced DL techniques have been documented for the identification and categorization of GMs using CT and MRI scans (117). CNNs, which are particularly adept at processing spatial information in images by learning hierarchical features through convolutional filters, have been widely employed for this task.

For instance, Chen et al. (118) developed a computation method called “GPS-OCM” to accelerate the investigation of metabolites associated with ovarian cancer. This method is based on the assessment of the similarity between metabolites and diseases. This method combines the techniques of GCN, principal component analysis (PCA), and SVM. The GCN was employed to extract network topology characteristics, while PCA was utilized to decrease the dimensionality of illness and metabolite variables. The SVM algorithm was utilized for the purpose of classification. The studies demonstrated the exceptional precision of our approach, as evidenced by the high values of AUC and Area Under the Precision-Recall Curve (AUPR).

Schwartz et al. (119) developed an automated methodology that aims to learn how to detect ovarian cancer in transgenic mice using optical coherence tomography (OCT) recordings. The process of classification is achieved by employing a neural network that is capable of perceiving spatially arranged sequences of tomograms. The authors introduced three neural network-based methodologies, including a feed-forward network backed by VGG, a three-dimensional (3D) convolutional neural network, and a convolutional long short-term memory (LSTM) network. Their experimental findings demonstrate that our models reach a favorable level of performance without the need for manual adjustment or the creation of specific features, despite the presence of severe noise in OCT images. The convolutional LSTM-based neural network, which is their most successful model, obtains a mean AUC of 0.81 ± 0.037 (standard error).

Tanabe et al. (120) sought to create a method called complete serum glycopeptide spectra analysis (CSGSA-AI) that uses AI and CNN to identify abnormal glycans in blood samples from patients with EOC. The researchers transformed the patterns of serum glycopeptide expression into two-dimensional (2D) barcodes in order to enable a CNN to learn and differentiate between EOC and non-EOC cases. The CNN model was trained using 60% of the available samples and validated using the remaining 40%. The researchers found that using principal component analysis-based alignment of glycopeptides to create 2D barcodes greatly improved the diagnostic accuracy of the approach, with a rate of 88%. By training a CNN with 2D barcodes that were colored according to the serum levels of CA125 and HE4, a diagnosis accuracy of 95% was attained. They are of the opinion that this uncomplicated and inexpensive approach will enhance the identification of EOC.

A comprehensive framework for detecting and classifying cervical cancer was created utilizing an optimized SOD-GAN (121). This advanced technique was designed to handle multivariate data sources. The suggested classifier accurately detects the cervix without the need for manual annotations or interventions. Additionally, it categorizes cervical cells as benign, precancerous, or cancerous lesions. The proposed approach has been expanded to include the identification of both the kind and stage of cervical cancer, in addition to its original purpose of diagnosing cervical cancer. Experiments were conducted during the training, validation, and testing phases of the proposed optimized SOD-GAN. Throughout all stages, the proposed approach demonstrated a high level of accuracy, reaching over 97% with a minimal loss of less than 1%. During the clinical analysis of 852 samples, the average duration required to classify the cervical lesion was 0.2 seconds. Therefore, the suggested method may effectively train the network through incremental learning, making it an ideal model for real-time cervical cancer diagnosis and prognosis.

The study conducted by Fekri-Ershad et al. (122) introduced a combination method that utilizes a machine learning approach. This method is characterized by a distinct separation between the feature extraction step and the classification stage. However, deep networks are employed during the feature extraction stage. This research introduces a neural network called a multi-layer perceptron (MLP), which is trained using deep features. The tuning of the number of hidden layer neurons is based on four novel concepts. In addition, MLP has been fed with ResNet-34, ResNet-50, and VGG-19 deep networks. In this technique, the layers responsible for the classification phase are eliminated in both CNN networks. The outputs then travel via a flattening layer before being fed into the MLP. To enhance performance, both CNNs are trained on correlated images utilizing the Adam optimizer. The proposed method was assessed using the Herlev benchmark database and achieved an accuracy of 99.23% for the two-class scenario and 97.65% for the seven-class scenario. The results indicate that the suggested method has achieved superior accuracy compared to both the baseline networks and other current methods.

Chandran et al. (123) presented two deep learning CNN structures for the identification of cervical cancer using colposcopy images. The first model is VGG19 (TL), while the second model is CYENET. The VGG19 model is utilized as a transfer learning technique in the CNN architecture for the research. A novel model, called the Colposcopy Ensemble Network (CYENET), was created to automatically classify cervical malignancies based on colposcopy images. The model’s accuracy, specificity, and sensitivity were evaluated. The accuracy of VGG19’s categorization was 73.3%. The findings obtained for VGG19 (TL) were relatively satisfactory. Based on the kappa score of the VGG19 model, it may be inferred that it falls into the intermediate classification group. The experimental results demonstrated that the suggested CYENET displayed a high level of sensitivity, specificity, and kappa scores, reaching 92.4%, 96.2%, and 88%, respectively. The CYENET model demonstrates an enhanced classification accuracy of 92.3%, surpassing the VGG19 (TL) model by 19%.

Takahashi et al. (124) introduced an AI-powered method that can automatically identify the areas impacted by endometrial cancer in hysteroscopic images. A total of 177 patients with a previous hysteroscopy were included in this study. Among them, 60 had a normal endometrium, 21 had uterine myoma, 60 had endometrial polyps, 15 had atypical endometrial hyperplasia, and 21 had endometrial cancer. Three widely used deep neural network models were utilized to implement machine learning techniques, while a continuity analysis method was devised to improve the precision of cancer detection. Ultimately, they examined whether precision could be enhanced by amalgamating all the learned models. The findings indicate that the diagnostic accuracy using the usual technique was approximately 80% (78.91%–80.93%). However, this accuracy improved to 89% (83.94%–89.13%) when utilizing the proposed continuity analysis. Furthermore, when integrating the three neural networks, the accuracy was above 90% (specifically, 90.29%). The sensitivity and specificity were 91.66% and 89.36%, respectively.

### 4.2 Tumor classification and typing

**Figure 7:**
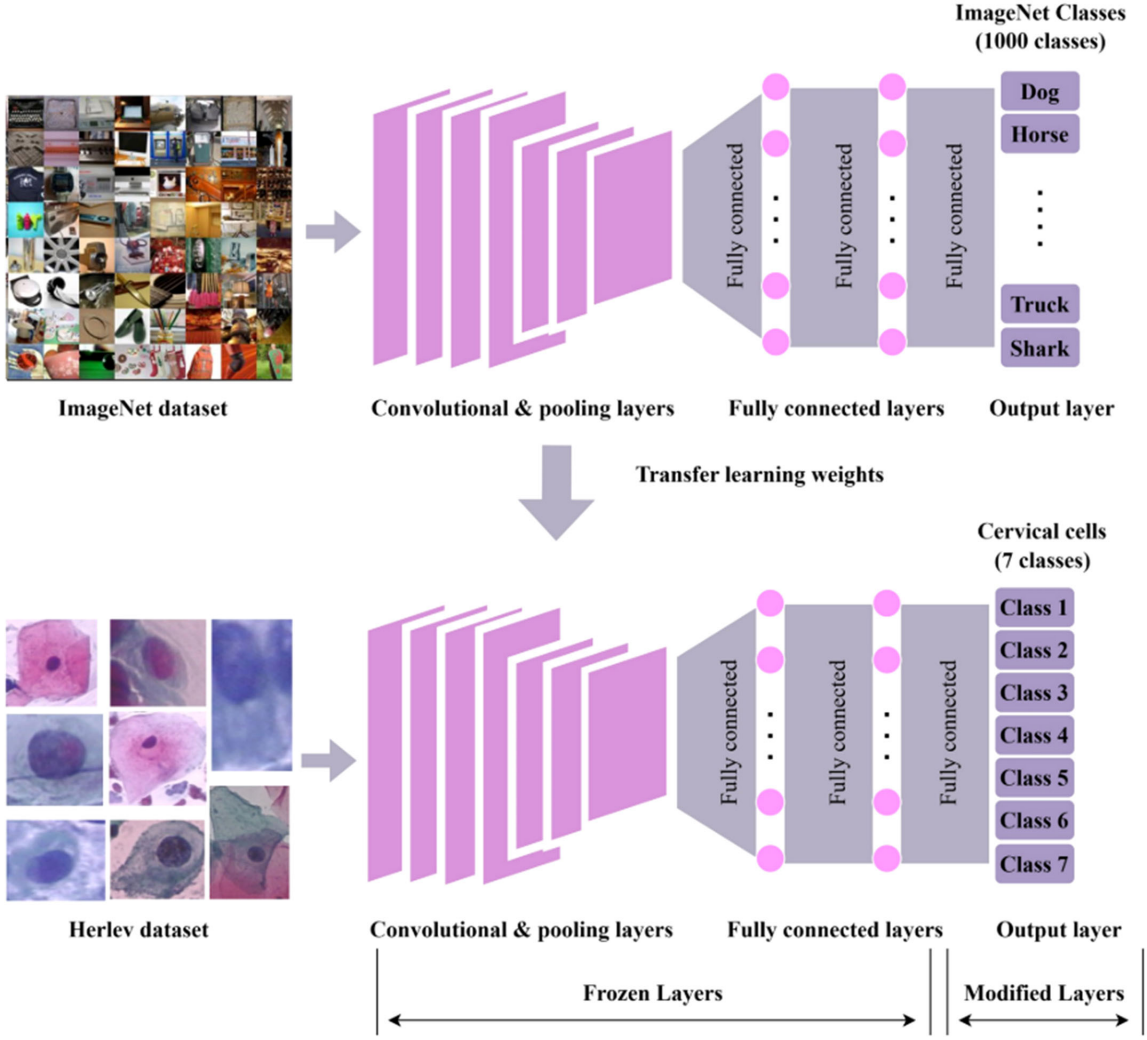
The workflow for cervical cancer classification using convolutional neural network (CNN) with transfer learning (126). Top: A CNN is pre-trained on the ImageNet dataset (containing 1,000 classes, e.g., Dog, Horse, Truck, and Shark), utilizing convolutional/pooling layers, fully connected layers, and an output layer to predict ImageNet classes. Bottom: Pre-trained weights are transferred to a new CNN for classifying seven classes of cervical cells from the Herlev dataset. In this transfer process, convolutional/pooling layers and most fully connected layers are frozen (kept unchanged), while only the output layer is modified to fit the seven cervical cell classes.

The process involves the detection and differentiation of non-cancerous and cancerous growths. In order to thoroughly assess GMs, whether they are benign or malignant, clinicians must first make an initial determination based on symptoms as well as laboratory and imaging tests. Currently, the most reliable method for distinguishing between benign and malignant GMs is the use of pathological analysis, through either a puncture biopsy or a postoperative pathological evaluation. However, the techniques used are invasive, and a puncture biopsy poses a specific risk of needle route metastases (125). Consequently, several studies have investigated the use of radiomics to detect both benign and malignant tumors (**Figure 7**). Here, the ability of AI to learn discriminative features directly from data is crucial. Deep Convolutional Neural Networks (DCNNs), such as the AlexNet-based architecture (57), which sparked the modern deep learning revolution by winning the ImageNet challenge, have been adapted for medical image analysis.

Wen et al. (127) utilized a novel 3D texture analysis technique to assess the structural alterations in the extracellular matrix (ECM) of various ovarian tissues, including normal ovarian stroma, high-risk ovarian stroma, benign ovarian tumors, low-grade ovarian serous cancers, high-grade ovarian serous cancers, and endometrioid tumors. The analysis was conducted using 3D second-harmonic generation (SHG) image data. Through the optimization of the number of textons, testing imaging weighting, and nearest neighbor numbers, they were able to attain high accuracy ranging from approximately 83% to 91% across different classes. This performance significantly surpassed that of the corresponding two-dimensional version. This application showcases the effectiveness of using quantitative computer vision evaluation of 3D SHG image features as a possible biomarker for assessing cancer stage and kind. Crucially, it does not depend on extracting basic fiber characteristics like size and alignment. This classification algorithm is a versatile technique that relies on pre-trained SHG images. It is particularly suitable for analyzing dynamic fibrillar characteristics in many types of tissues.

The study conducted by Wu et al. (128) utilized a DCNN based on AlexNet to autonomously categorize several forms of ovarian tumors from cytological images. The DCNN is composed of five convolutional layers, three max pooling layers, and two fully connected layers. Next, they trained the model using two sets of input data. The first set consisted of original image data, while the second set consisted of augmented image data that included image enhancement and image rotation. The testing findings are derived from the application of the 10-fold cross-validation technique, revealing that the accuracy of classification models has been enhanced from 72.76% to 78.20% by utilizing augmented photos as training data. The devised approach proved to be effective in categorizing ovarian tumors based on cytological images.

The study conducted by Liu et al. (129) focused on the development of a DL algorithm called the light scattering pattern-specific convolutional network (LSPS-net). This algorithm is integrated into a 2D light-scattering static cytometry system to enable automatic and label-free analysis of individual cervical cells. A classification accuracy of 95.46% was achieved for distinguishing between normal cervical cells and malignant cells (specifically, a mixture of C-33A and CaSki cells). When used to classify label-free cervical cell lines, the LSPS-net cytometric approach achieves an accuracy rate of 93.31%. Additionally, the three-way categorization of the aforementioned cell types achieves an accuracy rate of 90.90%. Comparisons with alternative feature descriptors and classification methods demonstrate the superior capability of deep learning for automatically extracting features. The LSPS-net static cytometry has the potential to be used for early screening of cervical cancer. This method is characterized by its rapidity, automation, and lack of labelling requirements.

The research by Ghoneim et al. (87) presented a system for detecting and classifying cervical cancer cells using CNNs. The cellular images were input into a CNN model in order to extract features that have been learned at a deep level. Next, an extreme learning machine (ELM)-based classifier was used to classify the input photos. The CNN model was employed using the techniques of transfer learning and fine-tuning. Additionally, the study explored other classifiers, such as MLP- and AE-based classifiers, in addition to the ELM. The Herlev database was used for conducting experiments. The CNN-ELM-based system demonstrated a detection accuracy of 99.5% for the two-class problem and a classification accuracy of 91.2% for the seven-class challenge.

The study conducted by Li et al. (130) aimed to develop an AI system capable of automatically identifying and diagnosing abnormal images of endometrial cell clumps (ECCs). The researchers used the Li Brush to collect endometrial cells from the patients. Slides were generated using the liquid-based cytology technique. The slides were digitized and categorized into malignant and benign groups. The authors put forward two networks, namely, a U-Net segmentation network and a Dense Convolutional Network (DenseNet) classification network, for the purpose of image identification. Four more categorization networks were utilized for comparative testing. We gathered a total of 113 endometrial samples, with 42 being malignant and 71 being benign. From these samples, we created a dataset consisting of 15,913 images. The segmentation network obtained a total of 39,000 patches of ECCs. Subsequently, a total of 26,880 patches were utilized for training, whereas 11,520 patches were allocated for testing. Assuming that the training set achieved a 100% success rate, the testing set achieved an accuracy of 93.5%, a specificity of 92.2%, and a sensitivity of 92.0%. The remaining 600 cancerous patches were used for verification. A successful AI system was developed to accurately categorize ECCs as either malignant or benign.

Retrospectively, clinical information and the most recent preoperative pelvic MRI were gathered from patients who had undergone surgery and were diagnosed with uterine endometrioid adenocarcinoma based on pathological examination. The region of interest (ROI) was subsequently delineated in T1-weighted imaging (T1WI), T2-weighted imaging (T2WI), and diffusion-weighted imaging (DWI) MR images. From these scans, both classical radiomic features and deep learning image features were recovered. A comprehensive radiomics nomogram model was developed by merging conventional radiomics features, DL image features, and clinical information (131). The purpose of this model is to accurately differentiate between patients at low risk and those at high risk, as per the 2020 European Society for Medical Oncology (ESMO)–ESGO–European Society for Radiotherapy & Oncology (ESTRO) criteria. The effectiveness of the model was assessed in both the training and validation sets. Utilizing MRI-based radiomics models can be advantageous in categorizing the preoperative risk of individuals diagnosed with uterine endometrioid cancer.

### 4.3 Image segmentation and volume computation

The most prevalent modalities for acquiring images are CT, MRI, positron emission tomography (PET), and ultrasound (132). Images acquired using identical machine equipment, scanning technique, and scanning layer thickness do not require post-processing during feature extraction. Nevertheless, images received through various equipment and under diverse acquisition conditions necessitate pre-processing prior to feature extraction. The pre-processing procedure involves resampling, standardization, and high-pass filtering in order to achieve a consistent layer thickness and matrix size for feature extraction.

Once medical images are acquired, a specific ROI is usually defined by a process that includes automatic segmentation, manual segmentation, and semi-automatic segmentation. Automated segmentation is efficient in defining lesions but lacks accuracy in recognizing them. Furthermore, the boundaries of tumors in medical images are often indistinct, and the presence of nearby metastases and accompanying symptoms, such as inflammation, can readily disrupt the contours produced by semi-automatic and automatic segmentation. Conversely, manual segmentation is a subjective and time-consuming process that relies on clinicians identifying the lesions and drawing their outlines. Semi-automated segmentation, derived from automatic segmentation, enables doctors to manually review and correct the delineated edges, hence enhancing the efficiency and accuracy of the delineation process (132). Presently, the standard software for ROI mapping comprises the MIM (www.mimsoftware.com), ITK-SNAP (www.itksnap.com), 3D Slicer (www.slicer.org), and ImageJ (National Institutes of Health) software.

Medical image processing has widely used CNNs, which have shown remarkable success in tasks like image classification and segmentation (30). Engineers specifically design CNNs (74) to capture spatial correlations in tasks like image classification, segmentation, and object detection. Transformers have recently gained prominence in the field of medical image processing, demonstrating promising outcomes in a variety of tasks. The primary benefit of transformers compared to CNNs lies in their capacity to effectively manage extensive dependencies and correlations among pixels within an image. Several regions of a medical image may exhibit interconnected characteristics that significantly impact the diagnosis or therapy process. Transformers, equipped with their own self-attention mechanism, can efficiently record these linkages and dependencies, resulting in enhanced performance in tasks like lesion categorization or segmentation. This self-attention mechanism allows for simultaneous processing, making transformers more efficient than CNNs and U-Nets. Transformers have the advantage of being trainable on large datasets, allowing them to acquire more intricate representations of medical images. Nevertheless, transformers exhibit suboptimal performance when confronted with a restricted dataset size. In medical imaging, the availability of large datasets is often limited, making this particularly important.

DL algorithms were utilized as a diagnostic tool for analyzing CT scan images of the ovarian area (133). The photos underwent a sequence of pre-processing procedures, and subsequently, the tumor was segmented using the U-Net model. The occurrences were subsequently categorized into two groups: benign and malignant tumors. The classification task was executed utilizing deep learning architectures like CNN, ResNet, DenseNet, Inception-ResNet, VGG16, and Xception, in addition to machine learning models such as Random Forest, Gradient Boosting, AdaBoosting, and XGBoosting. The DenseNet 121 model achieved the highest accuracy of 95.7% on this dataset after optimizing the machine learning models.

A CNN (134) was constructed for the categorization of image patches in cervical imaging, with the aim of detecting cervical cancer. Manually extracted image patches of 15 × 15 pixels were identified using a shallow-layer CNN. The CNN consisted of a single convolutional layer, a ReLU activation function, a pooling layer, and two fully connected layers. The patches belonged to both VIA-positive and VIA-negative areas. The shallow CNN model has a classification accuracy of 100%. Despite the intricate computations involved in training a CNN, once trained, it is capable of classifying a new image in nearly real time.

Zhang et al. (62) conducted a study where they utilized DL techniques to achieve accurate and efficient automatic segmentation and applicator reconstruction in planning CT for cervical cancer brachytherapy (BT). The researchers introduced a new design for a 3D CNN called DSD-UNET. The dataset consisting of 91 patients who had CT-based brachytherapy for cervical cancer was utilized to train and evaluate the DSD-UNET model for the automatic segmentation of the high-risk clinical target volume (HR-CTV) and organs at risk (OARs). Applicator reconstruction was accomplished through the use of DSD-UNET-based segmentation to identify the different components of the applicator. This was followed by the creation of a 3D skeleton and fitting a polynomial curve to it. An assessment was conducted on the digitization of the channel routes for the tandem and ovoid applicators during the planning of CT. This evaluation utilized data from 32 patients. The accuracy was statistically evaluated using the Dice similarity coefficient (DSC), the Jaccard index (JI), and the Hausdorff distance (HD). The segmentation performance of DSD-UNET was evaluated in comparison to that of 3D U-Net. The results demonstrated that the DSD-UNET method had superior performance compared to the 3D U-Net method in segmenting all of the structures.

The study conducted by Hodneland et al. (135) introduced a completely automated method for segmenting the primary tumor in endometrial cancer. This method utilizes three-dimensional convolutional neural networks and is applied to preoperative pelvic MR scans. Using this method, tumor volume estimates and segmentation accuracy achieved by CNNs are equivalent to those achieved through manual segmentation by radiologists. The use of CNN for tumor segmentation allows for automated and accurate identification of tumors. This technique opens up new possibilities for quickly analyzing the entire volume of a tumor and extracting radiomic features. These features can possibly be used to identify prognostic markers, which in turn may lead to more personalized treatment for patients with EC.

### 4.4 Gene mutation state and prediction

The human genome sequence can undergo alterations of varying sizes, including insertions, deletions, or inversions. These modifications can range from a single nucleotide base to an entire chromosome (136). Genetic alterations with a length greater than 1,000 bases characterize structural variants (137). Copy number variations (CNVs) and copy number alterations (CNAs) are big differences in the structure of DNA that can be found in 12% of human genomes (138). They are noteworthy due to their association with many illnesses.

Different harmful types of EOCs have gene mutations in different places. Approximately half of EOCs do not fix homologous recombination properly. Homologous recombination repair defects are primarily caused by mutations in the BRCA gene. This gene, acknowledged as a significant tumor suppressor gene (139), plays a crucial role in repairing DNA double-strand breaks during homologous recombination repair. Patients with advanced ovarian cancer who have BRCA1/2 mutations show enhanced responsiveness to platinum-based chemotherapeutic treatments. They also had better rates of objective remission and survival after treatment with platinum-based medicines. Additionally, giving poly(ADP-ribose) polymerase inhibitors to people who have OC after platinum-based chemotherapy can greatly lower their risk of recurrence and death (140). Importantly, by examining the H&E-stained pathological images of tumors, a DL model can detect genetic changes.

In the study conducted by Zhu et al. (141), they utilized the findings from this effort to identify a cluster of putative new genes associated with HPV infection in a protein–protein interaction network. The random walk with restart (RWR) method was utilized on the protein–protein interaction (PPI) network, with known genes associated with HPV infection serving as seed nodes. Following the application of the permutation test to filter out genes occupying specific positions in the PPI network, genes with strong interaction confidence and functional similarity to known HPV infection-related genes were chosen using the association test. This selection process involved consulting published databases such as STRING, gene ontology (GO) terms, and the Kyoto Encyclopedia of Genes and Genomes (KEGG) pathway.

Bahado-Singh et al. (142) utilized AI to detect the most influential epigenetic markers throughout the entire genome. Both logistic regression and AI methods consistently achieved good diagnostic accuracy in detecting less invasive ovarian cancer using cytosine methylation alterations in circulating cell-free DNA. Comparable findings were acquired for CpG markers limited to the promoter region, which is believed to be implicated in the initial stages of cancerous transformation. This work showcases the potential significance of precision oncology, which involves the integration of AI with epigenomic analysis. It proves that this combination can be used to accurately diagnose and understand the development of OC. The latter is crucial for the advancement and implementation of innovative targeted therapies, such as CRISPR-based DNA methylation.

The research by Guo et al. (143) introduced a model called lncRNA-disease associations by combining (LDACE), which utilizes a combination of Extreme Learning Machines (ELMs) and Convolutional Neural Networks (CNNs) to predict potential connections between lncRNAs and diseases using ML. More precisely, the representation vectors are formed by combining several types of biological information, such as functional similarity and semantic similarity. Next, the CNN is utilized to extract both local and global characteristics. Ultimately, ELM is selected to conduct the prediction task in order to identify potential correlations between lncRNAs and diseases. The proposed method demonstrated a notable area under the receiver operating characteristic curve of 0.9086 in leave-one-out cross-validation and 0.8994 in fivefold cross-validation.

### 4.5 Metastasis

The main purpose of preoperative imaging evaluation is to detect lymph node metastasis (LNM) by employing size criteria (≥10 mm in the short axis). However, this approach frequently has low sensitivity because it cannot distinguish normal-sized metastatic lymph nodes (144). Regrettably, a majority of metastatic lymph nodes in clinical practice measure smaller than 10 mm (145). This indicates that conventional imaging techniques have significant challenges in detecting normal-sized lymph node metastases.

Because of the rapid advancement of quantitative image analytics, researchers have shifted their reliance from solely visual indicators to concentrating on semantic features derived from image data (146). DL is considered a highly promising technology in the realm of medical imaging. DL has the ability to revolutionize image analysis by automatically discovering important feature representations for various tasks (17). DL has been utilized to forecast LNM utilizing various medical images from diverse types of tumors, including the prediction of LNM in normal-sized lymph nodes (147).

An evaluation was conducted to assess the efficacy of sparse-sampling CT with DL-based reconstruction in detecting metastasis of malignant ovarian tumors. Urase et al. (148) acquired contrast-enhanced CT scans (n = 141) of ovarian tumors from a publicly available database. These images were then randomly split into 71 training cases, 20 validation cases, and 50 test cases. The software simulation was used to calculate the CT images slice-by-slice using sparse sampling. The evaluation involved two deep learning models, namely, the Residual Encoder–Decoder Convolutional Neural Network (RED-CNN) and the deeper U-Net, which were used for deep learning-based reconstruction. They assessed the peak signal-to-noise ratio (PSNR) and structural similarity index measure (SSIM) as quantitative measurements for 50 test cases. Two radiologists conducted a separate qualitative assessment for the following criteria: overall quality of the CT image, clarity of the iliac artery, and visibility of peritoneal spread, liver metastasis, and lymph node metastases. The Wilcoxon signed-rank test was employed to compare the image quality of the two models, while the McNemar test was used to analyze the metastatic detectability. The average PSNR and SSIM exhibited superior performance when using a U-Net with greater depth compared to the RED-CNN model. In terms of visual evaluation, the deeper U-Net model outperformed the RED-CNN model in all aspects. The detectability of metastasis using a deeper U-Net model exceeded 95%. The utilization of deep learning-based reconstruction in sparse-sampling CT has demonstrated its efficacy in detecting metastases of malignant ovarian tumors and has the potential to decrease the overall radiation exposure associated with CT scans.

Qian et al. (149) conducted a study to construct a non-invasive DL nomogram model that uses RESOLVE-DWI and clinical information to predict the presence of normal-sized LNM in cervical cancer patients before surgery. The integrated model that incorporated RESOLVE-DWI and Analog-to-Digital Converter (ADC) maps demonstrated superior performance compared to the two models that relied solely on single-modality MR images. The DL nomogram, which incorporates the combination model along with age, tumor size, FIGO stage, ADC value, and Squamous Cell Carcinoma antigen (SCCa) level, demonstrated the highest level of performance. It achieved AUCs of 0.890 and 0.844 in the development and test cohorts, respectively.

Feng et al. (150) claimed that by analyzing histological images, AI can predict LNM in EC. The present study used a DL neural network technique to complete a binary classification task and forecast the existence or absence of lymph node metastasis in esophageal cancer. The model was validated using an independent cohort. EC encompasses a collection of diverse tumors with variations in their physical and cellular characteristics. Curettage specimens are insufficient for capturing the complete range of tumor features. Therefore, in their investigation, the researchers used pathological images of EC derived from paraffin-embedded tissues following surgical excision. This approach allowed for the optimal visualization of the morphological features of cancerous tissue as well as the acquisition of highly indicative information regarding lymph node metastasis. The most prominent DL features for predicting LNM are emphasized to provide pathologists with a clear understanding and to ensure transparency in the creation of the multiple instance learning (MIL) model.

### 4.6 Efficacy against chemotherapy and platinum resistance

**Figure 8:**
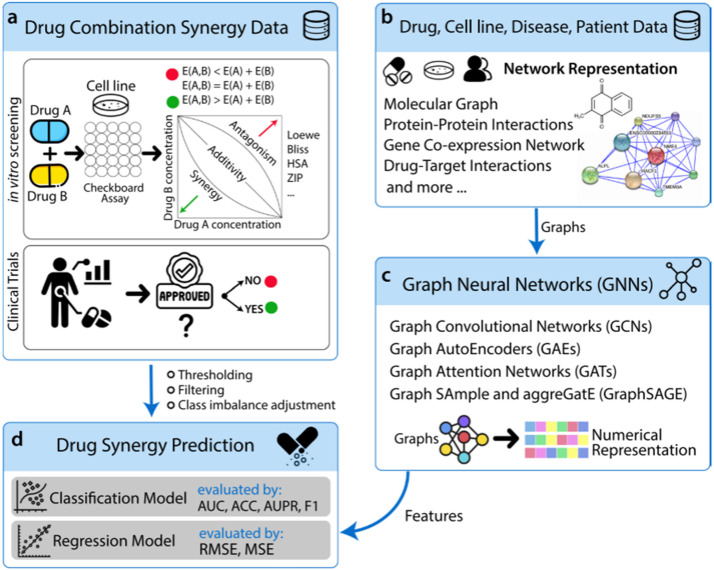
Schematic view of relevant data and the generic pipeline of synergistic drug combination prediction using GNNs (69). The pipeline includes **(a)** drug combination synergy data from *in vitro* screening and clinical trials, illustrating synergy assessment methods (e.g., Loewe and Bliss); **(b)** structuring multimodal data (e.g., molecular graphs and protein–protein interactions) into network representations; **(c)** utilizing graph neural networks (GNNs) to convert graph data into numerical feature representations; and **(d)** predicting drug synergy through classification or regression models and evaluating their performance using standard metrics (e.g., AUC, ACC, and RMSE). AUC, area under the receiver operating characteristic curve; ACC, accuracy; RMSE, root mean square error.

Despite significant progress in precision medicine, properly and proactively identifying platinum resistance in patients continues to be a challenging task. If a patient is expected to have a high likelihood of becoming resistant to platinum-based treatment, it is feasible to provide a more efficacious treatment than the standard of care, which often involves a combination of chemotherapy drugs, including platinum. For instance, we can optimize the strategy and timing of the surgical procedure for secondary cytoreductive surgery, thereby reducing the growth potential of the drug-resistant subclonal tumor population (**Figure 8**) (151). Simultaneously, people who are resistant to drugs may undergo more regular testing to quickly identify tumor recurrence. Also, platinum resistance is an easy way to find out how sensitive patients are to PARPi (152). As a result, making precise predictions of platinum resistance in patients will reduce the need for wasteful and burdensome clinical testing. Hence, precisely identifying platinum-resistant patients will enable them to fully leverage the advantages of precision medicine. Predicting treatment response requires models that can capture subtle, prognostic patterns in complex data. Deep learning models, particularly those leveraging PET/CT imaging, are adept at this.

Lei et al. (47) conducted a study that developed a comprehensive DL model to accurately predict the platinum sensitivity of patients diagnosed with EOC. It was very accurate and precise that the entire abdomen model, which used the whole abdomen as the volume of interest (VOI) on the axial CE-T1WI and T2WI sequences, predicted platinum sensitivity in people with EOC. Strong calibration and decision curves confirmed the validity of the model. Also, the algorithm did a good job of telling the difference between people who had a high chance of recurrence and those who did not, showing good accuracy in predicting progression-free survival (PFS) for 1 year. Furthermore, the heatmaps appeared to link the spatial arrangement of regions with significant levels of reaction to the susceptibility of platinum.

Yu et al. (153) created convolutional neural network models to analyze cellular patterns and morphology in a group of patients diagnosed with serous ovarian cancer. Their models effectively detected ovarian cancer cells, categorized histological grade and transcriptome subgroups, and forecasted patients’ reactions to platinum-based chemotherapy. They additionally performed differential expression and enrichment analyses to establish connections between the results of our quantitative histopathology research and the underlying biological pathways. Crucially, these methods rely solely on data and may easily adapt to include new types of tumors or the effectiveness of innovative treatment techniques. The advancement of these prediction systems will provide essential data for precision cancer care.

Zhuang et al. (154) provided a DL method for predicting platinum resistance in patients by analyzing their PET/CT medical images. Their solution provides enhanced detection efficiency in comparison to traditional methods, as it employs a comprehensive end-to-end workflow. The suggested deep model improves the accuracy of classification by combining the Squeeze-and-Excitation Block (SE Block) and the Spatial Pyramid Pooling Layer (SPPLayer) to group important data and conduct multilevel pooling. It was the most accurate at predicting platinum resistance in patients when the SE-SPP-DenseNet model was used. This model combines the DenseNet with the SE Block and SPPLayer.

### 4.7 Prognostic monitoring

DL algorithms have shown promise in forecasting the response to treatment in patients with gynecologic tumors using MRI data. Recent research has shown that CNNs may accurately forecast results, such as tumor reduction, local control, and overall survival, based on MR images taken before and after therapy for different types of cancer (155). Experts have looked into how multi-parametric MRI can be used to better predict how well different types of cancer will respond to treatment (156). This is performed by combining different imaging sequences.

Using DL techniques along with radiomics, the process of obtaining detailed information from medical images has made it more accurate to predict treatment response and prognosis (157). Treatment programs tailored to individual patients and improved tracking of patient progress are potential outcomes of this methodology. However, extensive, forward-looking studies have yet to confirm the practical usefulness of these models.

Using the ESTIMATE algorithms and the tumor-infiltrating immune cell (TIC) profile, Ma et al. (158) looked closely at the tumor microenvironment (TME) and found gene expression patterns in people who had EC. They found that TNFRSF4 is closely linked to the prognosis of EC and serves as a significant indicator of TME remodeling. Furthermore, they used clinical specimens to confirm the expression of TNFRSF4 in EC and nearby normal tissues, thereby supporting their findings. The study looked at the links between TNFRSF4 and immune-related markers like CD4, CD8, and FOXP3, as well as clinicopathologic features. Overall, their results suggest that TNFRSF4 could be used as a predictive biomarker because it plays a big role in the EC tumor microenvironment.

During the early stages, endometrial cancer does not show any symptoms. Additionally, there is a paucity of time-series correlation patterns that can help understand the transfer of clinical pathways, recurrence, and therapy for this illness. This study (159) evaluated the effectiveness of the artificial immune system (AIS) in conjunction with bootstrap sampling against a range of machine learning techniques, including both supervised and unsupervised learning approaches. The proposed method was compared with the backpropagation neural network and SVM using a radial basis function kernel, fuzzy c-means, and ant k-means to assess the sensitivity and specificity of the datasets. Additionally, the proposed method was used to predict the important factors of recurrent endometrial cancer.

Zhao et al. (160) conducted a retrospective study where they gathered data from 536 EC patients treated at Hubei Cancer Hospital between January 2017 and October 2022 and 487 EC patients from Tongji Hospital between January 2017 and December 2020 to use as an external validation group. The random forest model, the gradient elevator model, the support vector machine model, the artificial neural network model (ANNM), and the decision tree model were used to build the ovarian metastatic predictive model for EC patients. The effectiveness of five ML models was assessed using receiver operating characteristic curves and decision curve analysis. In order to identify possible predictors of ovarian metastasis in EC patients, factors such as tumor differentiation, lymph node metastasis, CA125, HE4, Alb, and LH can be utilized to develop a predictive model for ovarian metastasis in EC patients.

### 4.8 Drug synergism prediction

Combinations of cancer medicines can potentially provide therapeutic advantages by increasing the effectiveness of treatment and preventing resistance to single-drug therapy (161). Occasionally, the combined treatments may supply the individual pharmaceuticals at lower dosages compared to their use as single therapies. This approach helps to decrease the likelihood of treatment toxicity and other adverse effects. High-throughput screening (HTS) frequently assesses the phenotypic effects of drug combinations in preclinical cancer models to impartially investigate potential therapeutic combinations. Nevertheless, despite the use of automated HTS gear, the systematic screening of medication combinations has become difficult due to the exponential increase in the number of viable combinations. This is mostly due to the significant amount of time and patient specimens necessary for combinatorial testing. Moreover, the mechanisms that cause cancer to advance or develop resistance to treatment may vary significantly among patients, even if they have the same type of cancer. This variability presents additional experimental challenges, necessitating the examination of treatment combinations in the cells of each patient.

Recent studies have demonstrated that the DL model outperforms traditional machine learning algorithms in several biological applications (162). In order to achieve success in deep learning, high-quality datasets that contain experimental medication combinations are required. Due to advancements in high-throughput drug combination screening tests, the number of samples is increasing rapidly, resulting in a significant reduction in the data size limitation (163). DeepSynergy is an advanced prediction algorithm that uses deep learning to accurately identify synergistic medication combinations. The model has been trained using the dataset provided by Merck (164). Aside from its inadequate performance, this model is constrained in its interpretation due to the chosen methods for representing medications and cell lines, as well as the model’s architecture. It is challenging to determine the contribution or importance of drug descriptors, such as toxophores, physicochemical features, and fingerprints, in relation to the mechanism of drug action in cells using a feedforward neural network (165). The prediction of drug synergism is a cornerstone of modern precision oncology. The complexity of biological networks necessitates sophisticated AI models, a notion strongly supported by recent literature. As reviewed by Zheng et al. (166), graph-based deep learning approaches are particularly promising for this task, as they can effectively model the intricate relationships between drugs, targets, and cellular pathways, thereby accelerating the discovery of effective combination therapies for GMs.

GNNs are becoming increasingly popular in drug discovery due to their ability to efficiently process and analyze complex data, including molecular graphs and biological networks (167). There is abundant good evidence that GNN-based models can help with drug discovery. These models have performed well in virtual screening, predicting molecular properties, predicting protein–ligand binding, and repurposing drugs (168). GNNs have shown promise in finding connections and interactions in many areas (169), but it is still being studied how well they work in predicting how drugs will work together. Given the increasing use of GNNs in predicting drug synergy, their demonstrated effectiveness in comparison to widely used high-performing methods such as MatchMaker, DeepSynergy, and Deep Tensor Factorization (DTF), along with the growing significance of discovering drug combinations in both research and industry (170).

Computational techniques can improve the efficiency of drug combination screening. Despite recent advances in applying machine learning to synergistic drug combination prediction, several challenges remain. First, the performance of existing methods is suboptimal. There is still much room for improvement. Second, the model does not fully integrate biological knowledge. Finally, many models lack interpretability, limiting their clinical applications (171).

## 5 Deep learning of gynecologic malignancies based on pathological images

Histopathology constitutes a cornerstone of precision oncology. Histopathology or cytology must diagnose almost every type of solid tumor. Essentially, all clinical choices regarding treatment and follow-up rely on histological findings. In the field of digital pathology, high-resolution technology captures tissue slides in their entirety as whole slide images (WSIs). This process produces images with an extremely large number of pixels, commonly referred to as “gigapixel images” (21).

Recent advancements in pathology are currently propelling cancer diagnoses. Convolutional neural networks, which emerged in 2012, have consistently shown their ability to achieve high accuracy in classifying both medical and non-medical image datasets (59). Subsequently, a digital revolution in pathology commenced, involving the utilization of cutting-edge DL models developed by the computer vision community for analyzing digital histopathology slides. The H&E staining process is widely used in cancer diagnoses. There are well-established retrospective cohorts and clinical trial sets that are extensively characterized. These datasets allow for the creation of large-scale histopathology imaging datasets, which may be used to train advanced DL models. Several proof-of-concept studies have demonstrated the capacity of DL models to assist in the diagnosis and molecular classification of malignancies (172) as well as forecast patient prognosis (170) by identifying phenotypes on H&E-stained tumor slide images. A significant advantage of DL in pathology is its ability to process entire WSIs by breaking them down into smaller patches for analysis and then aggregating the results.

Deep neural networks have surpassed classical machine learning models in terms of classification accuracy (173). Nevertheless, these black boxes do not provide direct insight into the morphological characteristics they are associated with, which is a major concern for both mechanistic analysis and clinical decision-making (174). The presence of image artifacts, such as blurring, noise, and lossy image compression, may complicate the identification of morphological characteristics with biological significance (175). Studies have shown that tissue damage, image quality, and dataset-specific artefacts can influence the feature representation and prediction accuracy of neural networks (176). These artefacts significantly influence deep learning-based predictions, making it crucial to break down CNNs into biologically understandable features.

Sun et al. (177) devised a computer-aided diagnosis (CADx) method for analyzing histopathology images of endometrial disorders. Their methodology included a convolutional neural network and attention processes. The CADx method, known as HIENet, was proven to be successful in binary and multi-class classification tasks using a limited dataset of 3,500 H&E images in 10-fold cross-validation and external validation. It exhibited higher classification accuracies compared to three associate chief physicians. HIENet utilizes attention processes and a class activation map to effectively detect and emphasize morphological aspects in H&E images. This enhances the interpretability of the images, allowing pathologists to correlate pixel-level H&E image features with histological information. Given the aforementioned benefits, HIENet has the potential to be employed in a collaborative human–machine model for grading diagnosis in endometrial disorders, hence potentially enhancing the efficiency of pathologists.

Yiping Wang and colleagues (178) introduced a method that combines transfer learning and ensemble learning to automatically classify epithelial ovarian cancer WSIs. The suggested method outperformed both regular CNNs and pathologists without specialized training in gynecologic pathology across multiple performance criteria. These results indicate a potentially favorable future path to confirm the findings on a wider group of patients and investigate other deep learning structures that integrate characteristics from various levels of magnification and patch sizes. Moreover, as the majority of the improvement in performance is seen in the slide-level classification outcomes, we will evaluate the patch-level classification outcomes (such as five-dimensional feature vectors obtained from patch-level results) from different runs to gain a more comprehensive understanding of the modifications in patch-level classification that led to enhanced performance in slide-level classification.

A study by Song et al. (179) used WSIs of tissue slides to examine whether DL could be used to sort the different types of cervical and endometrial cancers into groups and find the exact location where adenocarcinomas start. For categorization, the WSIs were divided into image patches measuring 360 × 360 pixels at a magnification of ×20. Subsequently, the mean of the patch classification outcomes was employed for the ultimate categorization. The Area Under the Receiver Operating Characteristic Curve (AUROCs) for the cervical and endometrial cancer classifiers were 0.977 and 0.944, respectively. The adenocarcinoma origin classifier achieved an AUROC of 0.939. The results unequivocally showed that DL-based classifiers can effectively distinguish between cervical and uterine malignancies, proving their practicality.

Riasatian et al. (180) introduced a novel network, KimiaNet, in their study. This network utilizes the DenseNet topology, consisting of four dense blocks, and is fine-tuned and trained using histopathological images in various configurations. The researchers employed over 240,000 image patches, each consisting of 100 × 100 pixels, obtained at a magnification of ×20 using their novel “high-cellularity mosaic” technique. These patches were utilized to leverage weak labels for 7,126 whole slide images of formalin-fixed paraffin-embedded human pathology samples, which are publicly accessible through The Cancer Genome Atlas (TCGA) repository. The efficacy of search and classification in KimiaNet was evaluated by utilizing three public datasets: TCGA, endometrial cancer images, and colorectal cancer images. This evaluation involved testing the effectiveness of using different networks’ features for image representation. In addition, they developed and trained several convolutional batch-normalized ReLU (CBR) networks. The results show that KimiaNet is a better feature extractor for histopathological images than both the original DenseNet and smaller CBR networks.

## 6 Deep learning of gynecologic malignancies based on other omics

The genome of a tumor contains distinct molecular features that are exclusive to that tumor (181). Clinical genomics research is essential for achieving precision oncology because it investigates the human genome, specifically focusing on a disease’s genotype. As a result, the genotypic characteristics of the tumor, such as genomic instability or mutation status, add to the phenotypic and geographic alterations examined in histopathology. Clinical genomics utilizes various types of genomic data, including whole genome or exome sequencing, RNA sequencing, methylation assays, and copy number variation analysis, as sources of information. This technology enables the precise classification of the patient’s specific cancer type, potential origin, sensitivity to specific medications, and prognosis.

In the past, the analysis of genomic data was only carried out by conventional bioinformatics, which utilized algorithms to execute tasks such as sequence alignment, variant calling, or differential expression analysis. However, human specialists extensively craft these algorithms by hand, prioritizing the identification of predetermined patterns. AI’s potential benefit in clinical genomics lies in its ability to enhance the existing toolbox by enabling more comprehensive data analysis than was previously possible. Unlike vision-based models, DL applications in genomics often utilize different architectures, such as fully connected DNNs for tabular omics data or specialized models for sequence data. ML (182) has been essential in uncovering hidden or imperceptible patterns, such as the intricate folding of proteins or the distinctive markers resulting from mutagenesis events in our DNA. Traditional bioinformatics methods cannot achieve the potential of AI to uncover new ways of thinking that could lead to advancements in clinical genomics. One notable difference between AI in clinical genomics and histopathology is the wide range of model types employed. Computer vision derived DL models for histopathology, but computer science did not directly develop DL models for genomics. This led to the exploration of a wider range of model types in genomics.

The TME of ovarian cancer consists of many types of cells, including tumor cells, stromal cells, and immune cells, which have the ability to control the growth and advancement of the tumor (183). The presence of immune cell types, such as tumor-associated macrophages or Tumor-Infiltrating Lymphocytes (TILs), inside the TME has been demonstrated to influence both cancer prognosis and the response to neoadjuvant chemotherapy (NACT) (184). Previous studies on high-grade serous ovarian cancer (HGSOC) have primarily used tumor samples that consist of a mixture of different cell types with varied proportions (185).

Teng et al. (186) evaluated the effectiveness of matched transcriptome and proteome data obtained from a fabricated admixture series of HGSOC tumors, stroma/fibroblasts, and immune cells. Their study utilized existing deconvolution and prognostic molecular subtype prediction techniques. They conducted additional research to examine the influence of cell type combinations on the association between protein and transcript abundances. In various independent cohorts of patients with HGSOC, the authors presented optimized protein signatures for tumor, stroma, and immune cell mixes and evaluated their effectiveness in categorizing proteome data from enriched and bulk tissue collections.

The integration of multi-modal data (e.g., imaging, genomics, and clinical records) represents a frontier in predictive oncology, as highlighted in recent comprehensive reviews. For instance, the work by Tan et al. (187) underscored the potential of deep learning to unravel complex patterns across disparate data types, which is crucial for advancing personalized therapy in GMs.

## 7 Comparison of deep learning and conventional imaging techniques in imaging gynecologic malignancies

Although DL approaches have demonstrated potential in several GM imaging tasks, it is important to carefully evaluate the specific scenarios where traditional imaging techniques are still useful and those where DL tools are more suitable.

### 7.1 Traditional methods

Prior to the advent of deep learning, the analysis of medical images for gynecologic malignancies predominantly relied on traditional machine learning and image processing techniques. These methods typically involve a multi-stage pipeline: initial image pre-processing (e.g., noise reduction and normalization), followed by manual or semi-automated segmentation of ROIs. Subsequently, hand-crafted features—quantitative descriptors of shape, intensity, texture, and other statistical properties—are extracted from these ROIs. Finally, these features are used to train classical machine learning classifiers (e.g., support vector machines and random forests) for tasks such as detection, classification, and prognosis prediction. The following sections discuss the inherent advantages and limitations of these established methodologies.

#### 7.1.1 Advantages of traditional imaging techniques

Interpretability: Conventional imaging approaches produce easily understandable data by relying on established characteristics and manually designed statistical procedures (188). The interpretability of the results enables physicians to gain a deeper understanding of the reasoning behind the decision-making process. Establishing trust in the results and taking appropriate clinical actions require this understanding.

Reduced computational requirements: Conventional imaging methods often have fewer computational needs in comparison to deep learning approaches (189), rendering them more accessible and simpler to execute on regular workstations without the necessity for high-performance computer resources.

Robustness to changes: Traditional imaging methods are better at handling changes in imaging protocols and acquisition parameters (190) because they rely on well-known features that do not change as easily when it comes to image quality and appearance.

#### 7.1.2 Disadvantages of traditional methods

Monitoring systems or standard image processing techniques often identify candidate lesion areas in conventional computer-aided tumor diagnosis methods. The process of localizing a lesion involves multiple stages and typically relies on a substantial number of manually designed characteristics. The classifier is used to map eigenvectors to potential candidates in order to determine the probability of authentic lesions. Computer-aided diagnosis is a field of medical image analysis that can be improved (191). The classic approach uses the image’s pixel data to identify various visual elements, such as corners, contours, and color gradients, using a pre-defined formula. Various algorithms exhibit varying levels of accuracy in detecting these features, and distinct experimental methodologies also employ their own techniques for feature extraction. When an image undergoes linear or non-linear transformations, such as scaling, rotation, translation, affine transformation, or deformation, it may result in interference during feature extraction. Hence, contemporary and more sophisticated convolutional neural network algorithms surpass previous algorithms in specific features or total accuracy. Various algorithms possess varying capacities to handle these transformations, and the greater the problem-solving capability, the higher the level of robustness.

### 7.2 Deep learning in medical image analysis

#### 7.2.1 Performance improvements over traditional methods

DL approaches used in medical image analysis have yielded promising outcomes in different areas, resulting in notable progress in illness identification and diagnosis, anatomical structure segmentation, and treatment outcome prediction (189). DL algorithms have the ability to acquire intricate patterns from medical images, demonstrating strong adaptability to new data and obtaining performance that is comparable to or surpasses human capabilities in various tasks (192). This capability has the potential to significantly enhance the precision, effectiveness, and uniformity of medical image analysis, eventually boosting the quality of patient treatment and results.

Comparisons between deep learning models and older pre-deep learning imaging techniques have shown significant enhancements in performance. DL models have been successful in diagnosing diabetic retinopathy from retinal images, with a sensitivity of 96.8% and a specificity of 87.0%. These results are much better than those achieved by classical approaches, which had sensitivities ranging from 49.3% to 85.5% and specificities ranging from 71.0% to 93.4% (193). Deep learning-based detection of pulmonary nodules on CT scans has demonstrated a greater accuracy rate of 94.2% compared to conventional computer-aided detection approaches, which achieved an accuracy rate of 79.1% (194). Deep learning models have demonstrated superior performance in segmenting brain tumors from MRI scans, achieving a Dice similarity coefficient of 0.88. In comparison, standard approaches have yielded coefficients ranging from 0.65 to 0.85 (195).

In addition, DL has facilitated the creation of models that can combine several types of imaging data at different scales, along with clinical and demographic information, in order to produce more precise and comprehensive forecasts on patient outcomes and responses to therapy (192). This capability has played a significant role in the expanding field of radiomics, which seeks to extract and analyze complex quantitative characteristics from medical images in order to develop predictive models for personalized treatment (114). DL is becoming more and more critical in the advancement of precision medicine and the improvement of patient care for a variety of diseases and medical conditions.

#### 7.2.2 Controversies and disparities

A significant obstacle in the domain of DL for imaging GMs is the absence of standardization and benchmarking. Various studies have utilized a range of datasets, preprocessing methodologies, model architectures, and training strategies, which complicates the comparison of model performance and the evaluation of their clinical usefulness (196). The lack of standard reporting of model performance parameters, such as accuracy, sensitivity, and specificity, makes it more difficult to compare results across different studies. Future research should prioritize the establishment of standardized datasets, evaluation measures, and reporting requirements to simplify the process of benchmarking and comparing DL models. Also, supporting open research methods like sharing data and code could speed up the development and validation of deep learning models for imaging GMs.

The majority of DL research in the field of GM imaging has concentrated on analyzing data from single imaging modalities, such as MRI, CT, or PET scans. By incorporating multimodal information, such as merging functional and anatomical imaging data, it is possible to enhance the effectiveness of DL models and gain a more thorough understanding of tumor characteristics (197). Furthermore, the inclusion of temporal data derived from longitudinal imaging data has the potential to improve the accuracy of predicting treatment response, tumor recurrence, and patient outcomes (198). Integrating clinical information, including patient demographics, tumor histology, and treatment details, can enhance the effectiveness of DL models in managing GMs, in addition to imaging data. Further investigation is needed to examine the advancement of DL models that can efficiently include multimodal, temporal, and clinical data for personalized therapy planning and prognostication.

DL models, namely, CNNs, are commonly regarded as “black boxes” because of their intricate structures and the absence of transparency in the decision-making process (165). The absence of transparency can impede the use of DL methods in clinical practice, as physicians may be hesitant to rely on a model’s predictions without comprehending the underlying rationale. The development of DL models that are explainable and interpretable is essential in order to close this gap and encourage their acceptance in the medical community (199). Methods such as attention processes, layer-wise relevance propagation, and visualization of feature maps can be used to clarify the reasoning behind a model’s predictions and establish trust among doctors. Future research should prioritize the integration of explainability and interpretability into the development of DL models for imaging GMs. Additionally, efforts should be made to create techniques for evaluating the reliability and resilience of these models when dealing with noisy, incomplete, or adversarial data.

In order to implement DL models in clinical practice, it is necessary to thoroughly validate and quantify their effect on patient outcomes (200). Conducting extensive and future-oriented investigations would be valuable in determining the effectiveness, applicability, and practicality of DL models for imaging head and neck cancer (201). These studies should include varied patient groups and imaging data from several centers to ensure the strength and reliability of the models in real-world scenarios. Furthermore, it is crucial to incorporate these models into current clinical workflows, analyze their cost-effectiveness, and assess their impact on patient care. This includes reducing diagnostic errors, optimizing treatment planning, and enhancing patient outcomes. These steps are necessary for the successful implementation of these models (202). Further investigation should also focus on creating user-friendly, scalable, and secure software tools and platforms that can assist in the implementation of DL models in clinical settings and promote their broad usage in the therapy of GMs.

## 8 Challenges of deep learning models in the diagnosis and management of gynecologic malignancies

AI can play multiple important roles in gynecologic imaging, beyond just screening and detection. These roles include helping radiologists make accurate diagnoses, assisting clinicians in developing effective treatment plans, and incorporating various clinical–pathological–immunohistochemical factors to predict the likelihood of recurrence or metastasis. Therefore, it is anticipated that AI in gynecologic imaging will play a significant role in advancing precision medicine and tailored treatment. Nevertheless, there are several complex technical and clinical obstacles that hinder the sustainable progress of AI in gynecologic imaging.

### 8.1 Technical challenges

ML algorithms typically necessitate extensive datasets to achieve adequate performance. However, the clinical data available for diagnosing cervical cancer are frequently constrained in terms of both size and quality. In order to overcome these restrictions, researchers have employed many techniques for data pre-processing, including data augmentation, image enhancement, and the development of image-generating tools. These methods have been used to tackle issues such as uneven class distribution and short datasets. Nevertheless, a comprehensive approach is still necessary to tackle this problem.

A major issue in the development and implementation of DL systems in healthcare is the presence of intrinsic biases in many datasets. This bias can be based on factors such as ethnicity, sex, socio-economic circumstances of participants, or the institution where the studies were conducted (203). Therefore, it is necessary to implement more equitable and varied methods of collecting data for future investigations. This, in turn, will improve the overall applicability of deep learning models once again. Furthermore, it is necessary to develop national and international standards for data curation, especially when dealing with homogenous data, in order to ensure the comparability of data across different institutions. In addition, as changes may occur within the populations to which AI is applied, there is a need for model updates and reconfigurations. This aspect is often overlooked in the current model design (204). This will ultimately enable the acquisition of deep learning models that can learn dynamically during deployment rather than being fixed after a single static training iteration.

In ML, models need a substantial amount of data in order to achieve proficiency in their designated activity. One reason for this necessity is due to technical constraints, as multiple repeats of patterns are necessary to manipulate the internal model parameters into their intended state. Furthermore, the presence of variability in every biological system is another factor that necessitates data requirements. Tumors exhibit significant diversity in terms of their genetics, phenotype, and clinical behavior, which may vary between people. The training data collection must be of a minimum size that is sufficient to accurately represent the biological diversity. Consequently, studies with a small number of participants are unlikely to offer a wide range of data that can be applied to other datasets, especially in a clinical setting (205). Therefore, in order to ensure that DL models can be used in various clinical environments, it is necessary to obtain and distribute increasingly larger datasets. The primary limitation in training deep learning solutions for cancer research and oncology lies in the collection of data rather than the flexibility of the models. Histopathology, being the foundation of diagnosis, is more easily accessible compared to genetic data, which are usually expensive and not regularly obtained for all patients. As a result, it is more challenging to create genomic cohorts, especially for multi-omic methods. Well-funded research centers or large healthcare facilities are typically the only places where extensive clinical setups and infrastructure are available. An effective approach to tackling these difficulties is by employing distributed learning methods such as federated or swarm learning. These methods allow peers who are restricted from sharing public data to collaboratively train models (206). Moreover, technical concepts have the potential to enhance the process of gathering data. Techniques such as class balancing or boosting datasets with simulated samples can be beneficial for studies that have a limited number of patients (207). Conversely, enhanced ML models have the potential to be more efficient in their use of data and capable of learning effectively from smaller datasets. This could address the issue of limited data availability by employing a different approach (208).

DL models have a tendency to overfit, particularly when trained on small datasets, resulting in limited ability to generalize to new data (157). Overfitting can lead to the development of models that exhibit high performance on the training data but are unable to reliably forecast outcomes for new patients, hence restricting their therapeutic usefulness. When creating DL models for head and neck cancer imaging, it is important to consider regularization approaches and model architectures that can help prevent overfitting. Some examples of these strategies include dropout, batch normalization, and transfer learning. Furthermore, the use of cross-validation and external validation cohorts can aid in assessing and enhancing the generalizability of the model.

The training and deployment of DL models often require substantial computational resources, such as graphics processing units (GPUs) and specialized hardware (157). This need may pose a challenge for small clinical centers and researchers with limited access to high-performance computing facilities. Developing efficient model architectures, exploring strategies for model compression and acceleration, and utilizing cloud-based platforms for training and deployment can help to overcome these challenges and make DL models more accessible to a broader range of institutions and researchers.

### 8.2 Clinical challenges

Additional crucial factors must be stated, such as the clarity of the systems, which is linked to the data used, while the dependability, operation, and limitations of a single model must be evaluated. Additionally, it is important to consider additional variables such as ethical and medico-legal concerns, as well as the necessity for comprehensive validation and integration of AI systems into the current framework for clinical decision management, which has not yet been fully addressed. One important point to consider is whether AI can replace physicians in the activities of seeing, characterizing, and quantifying, which they currently perform using their cognitive abilities. The answer to this issue is likely to be negative. It is important to emphasize that the ultimate choice in patient diagnosis still lies with the physicians, not AI systems, and they bear the responsibility for it. One significant obstacle to the use of AI in clinical practice is the automation bias (209), which refers to the inclination to favor a diagnosis provided by a machine over the evidence based on scientific knowledge and the physician’s skill.

Physicians should possess a comprehensive understanding of how to effectively utilize and interpret AI algorithms in their practice. This includes discerning the appropriate scenarios in which a medical AI should be employed and determining the level of trust that should be attributed to the algorithmic conclusions. While AI presents new opportunities, the fundamental principles of clinical reality remain unchanged. To achieve significant influence on patient care, AI-based research in medical activities must adhere to the fundamental principles of medical science. Research hypotheses, whether based on AI or not, must adhere to clinical standards and be able to be justified in the clinical field (210). Medical AI must be trained in suitable contexts, utilizing optimized approaches and complete datasets, due to the ongoing development of evaluation tools in this challenging field (211). Furthermore, the integration of AI systems into large-scale data networks gives rise to legal and ethical issues relating to obtaining patient-specific consent, safeguarding privacy, sharing data, and ensuring multi-layered access to health information that is either fully or partially anonymized (212).

Currently, AI techniques lack transparency in their elaboration processes, meaning that their users may not have clear representations of how AIs have arrived at a specific conclusion. This lack of transparency can lead to “trust issues”, particularly when important decisions need to be made based on these conclusions. Furthermore, future research on the ethical integration of AI in medical assessment should take into account patients’ perceptions of these tools and determine the circumstances under which patients may feel neglected by their doctor due to the use of autonomous technologies for health recommendations and treatment (213). Ultimately, substantial volumes of data pertaining to GMs have been amassed and are rapidly expanding. Utilizing ML and DL techniques can enhance our understanding of the mechanisms behind GMs and improve the care of patients with these conditions. In the foreseeable future, the involvement of AI in decision-making processes is anticipated to increase. This is because AI systems possess desirable attributes such as the capacity to carry out uncomplicated but repetitive and time-consuming tasks, as well as the ability to optimize workflow management. By doing so, AI systems free up more time for clinical patient supervision (214).

## 9 Future directions of deep learning in gynecologic malignancies

In the future, neural networks can be applied in the medical field in two main ways: automatic diagnosis and assistance for doctors. Presently, less than one doctor per 1,000 people is available in 45% of WHO member nations. There is a high need for automatic diagnostic systems that utilize neural networks to evaluate patients without any risks. These systems help prevent clinicians from becoming overwhelmed and provide a clear schedule for patient visits. Automatic diagnosis is feasible in various imaging specialties such as X-rays, fluoroscopes, ultrasonography, CT, and MRI. These specialties focus on prevalent and debilitating diseases that affect the elderly population, including cardiovascular disease, cerebellomedullary diseases, and oncological diseases. These diseases are significant public health concerns. They will spearhead the advancement of less invasive techniques such as interventional radiology, interventional cardiology, and interventional neuroimaging (215).

Another prospective avenue is forecasting a medical occurrence, which allows a physician to discern the specific area that requires immediate attention. If the doctor predicts that a patient may need to visit again, they may schedule an early hospital appointment to prevent the symptoms from worsening. One illustration is the use of electronic medical data to assess occurrences such as symptoms, drug details, and appointment timetables. The neural network was trained using electronic medical records from 260,000 patients and 2,128 physicians over an 8-year span to determine the specific data and purpose for future medical evaluations. The recall function was employed in the approach, with a proportion of 79.58%. The training phase utilized 85% of the data, while the testing phase used 15% (216). Evaluating the prognosis is crucial for designing suitable therapy and follow-up approaches, which can either lead to patient recovery or extend their lifespan. Neural networks have demonstrated superior predictive ability in determining the survival rates of patients with several types of cancer, including breast, colorectal, lung, and prostate cancer, compared to other methods used in the area.

Future work will incorporate DL approaches for the diagnosis of all diseases, considering noise removal from any given dataset. The additional aspects and properties of DL models for medical images can be explored. To increase the accuracy, an enormous amount of data is required; therefore, the potential of the model should be improved to deal with large datasets. Also, different data augmentation techniques along with the required features of the dataset can be explored to attain better accuracy.

To enhance the validity of any models, it is imperative to include significantly larger cohorts from multiple centers and countries in future studies. The utilization of AI models in clinical settings is primarily focused on thyroid disorders, breast diseases, and liver diseases. Research on applying AI models to other systems is still largely in the theoretical phase. These clinical models should be utilized in future clinical prospective research to aid doctors in diagnostic and prognostic evaluations. It is necessary to summarize the difficulties and impacts that clinicians face while using artificial intelligence models and to continuously optimize these models. The progress in AI-based methodologies will enhance the precision of diagnoses, expedite the diagnostic procedure, and have a crucial function in aiding clinicians in decision-making and intelligent monitoring in the future (4).

Future endeavors will entail employing innovative approaches to tackle the scarcity of medical data. Techniques such as transfer learning and GANs can enhance smaller datasets, making them more comprehensive and resilient (49). These projects will increasingly rely on multidisciplinary teamwork. The UK’s Topol Fellowship provides healthcare professionals with an opportunity to acquire practical expertise in data science and AI, successfully closing the gap between two essential fields.

## 10 Conclusion

This review synthesized the application of AI across multi-omics data for gynecologic malignancies, providing an integrated examination of both technical methodologies and their clinical utility in diagnosis, prognosis, and therapy. Compared to existing literature, this work offers a more cohesive framework linking diverse AI models to specific clinical endpoints in GMs. Nevertheless, it also underscores critical shortcomings pervasive in the field. Most studies remain constrained by retrospective, single-center data; a pervasive lack of standardization in data processing, model development, and performance reporting hinders reproducibility and clinical translation—a challenge not always comprehensively addressed in previous reviews. Moving forward, overcoming these limitations necessitates a focused shift toward prospective, multi-institutional validation and the development of explainable AI (XAI) frameworks to bridge the gap between technical accuracy and clinical trust. The ultimate integration of AI into GM management depends on creating robust, interpretable, and clinically actionable tools that can be seamlessly adopted into real-world practice. The challenges and future perspectives discussed herein, such as data standardization and the need for XAI, are not unique to GMs but resonate with the broader field of AI in oncology, as highlighted in recent pan-cancer reviews (217).

In summary, AI has emerged as a powerful tool for handling large-scale datasets and is widely used to develop various omics models for GMs. Multi-omics analysis, which encompasses many techniques such as imaging, pathomics, genomics, metabolomics, and proteomics, has shown promise in improving the precision of diagnosing GMs. It can also aid in distinguishing between benign and malignant cases, as well as predicting the specific disease types and prognosis. Integrating multi-omics data has the potential to enhance patient survival and enable precision treatment in the future.

DL methods have made remarkable progress in the area of imaging for GMs. They have shown outstanding performance in important tasks like identifying tumors, segmenting them, and predicting outcomes. These techniques utilize different imaging modalities, such as MRI, CT, and PET scans. Integrating radiogenomics into DL models shows promise for advancing our understanding of tumor biology and heterogeneity, as well as directing personalized therapy regimens in the management of GMs.

Although the results are encouraging, there are several problems that need to be overcome in order to effectively employ DL models in the diagnosis and treatment of GMs. A significant challenge is the absence of uniformity in datasets, preprocessing methodologies, and model architectures, making it challenging to compare the performances of different models and evaluate their clinical usefulness. Obtaining extensive, varied, and thoroughly annotated datasets for imaging GMs is difficult because of privacy concerns, limitations on data sharing, and the time-consuming process of manually annotating by experts. Given the drawbacks of conventional imaging methods, such as the subjective and variable nature of human interpretation, combining DL with standard imaging techniques has the potential to enhance the reliability and precision of diagnosis and treatment planning. It is crucial to acknowledge the interdependent functions of these methods in order to make progress in the imaging and treatment of GMs.

Ultimately, it is crucial to carry out thorough clinical validations using extensive, forward-looking investigations in order to establish the effectiveness, applicability, and practicality of DL models for imaging GMs. To achieve better patient outcomes and implement personalized treatment strategies for managing GMs, it is crucial to overcome these obstacles and effectively combine advanced DL techniques with traditional imaging approaches.

## References

[1] Wiens J Shenoy ES. Machine learning for healthcare: on the verge of a major shift in healthcare epidemiology. Clin Infect Dis. (2018) 66:149–53. doi: 10.1093/cid/cix731, PMID: 29020316 PMC5850539

[2] Davenport T Kalakota R. The potential for artificial intelligence in healthcare. Future Healthc J. (2019) 6:94–8. doi: 10.7861/futurehosp.6-2-94, PMID: 31363513 PMC6616181

[3] Iqbal MJ Javed Z Sadia H Qureshi IA Irshad A Ahmed R. Clinical applications of artificial intelligence and machine learning in cancer diagnosis: looking into the future. Cancer Cell Int. (2021) 21:270. doi: 10.1186/s12935-021-01981-1, PMID: 34020642 PMC8139146

[4] Wang Y Lin W Zhuang X Wang X He Y Li L. Advances in artificial intelligence for the diagnosis and treatment of ovarian cancer (Review). Oncol Rep. (2024) 51:46. doi: 10.3892/or.2024.8705, PMID: 38240090 PMC10828921

[5] LeCun Y Bengio Y Hinton G. Deep learning. Nature. (2015) 521:436–44. doi: 10.1038/nature14539, PMID: 26017442

[6] Kermany DS Goldbaum M Cai W Valentim CCS Liang H Baxter SL. Identifying medical diagnoses and treatable diseases by image-based deep learning. Cell. (2018) 172:1122–31. doi: 10.1016/j.cell.2018.02.010, PMID: 29474911

[7] Zhang M Young GS Chen H Li J Qin L McFaline-Figueroa JR. Deep-learning detection of cancer metastases to the brain on MRI. J Magn Reson Imaging. (2020) 52:1227–36. doi: 10.1002/jmri.27129, PMID: 32167652 PMC7487020

[8] Abdel Razek AAK Alksas A Shehata M AbdelKhalek A Abdel Baky K El-Baz A. Clinical applications of artificial intelligence and radiomics in neuro-oncology imaging. Insights Imaging. (2021) 12:152. doi: 10.1186/s13244-021-01102-6, PMID: 34676470 PMC8531173

[9] Kann BH Hosny A Aerts HJWL. Artificial intelligence for clinical oncology. Cancer Cell. (2021) 39:916–27. doi: 10.1016/j.ccell.2021.04.002, PMID: 33930310 PMC8282694

[10] Lalehzarian SP Gowd AK Liu JN. Machine learning in orthopaedic surgery. World J Orthop. (2021) 12:685–99. doi: 10.5312/wjo.v12.i9.685, PMID: 34631452 PMC8472446

[11] Turkbey B Haider MA. Deep learning-based artificial intelligence applications in prostate MRI: brief summary. Br J Radiol. (2022) 95:20210563. doi: 10.1259/bjr.20210563, PMID: 34860562 PMC8978238

[12] Shin HC Roth HR Gao M Lu L Xu Z Nogues I. Deep convolutional neural networks for computer-aided detection: CNN architectures, dataset characteristics and transfer learning. IEEE Trans Med Imaging. (2016) 35:1285–98. doi: 10.1109/TMI.2016.2528162, PMID: 26886976 PMC4890616

[13] Beheshti I Ganaie MA Paliwal V Rastogi A Razzak I Tanveer M. Predicting brain age using machine learning algorithms: A comprehensive evaluation. IEEE J BioMed Health Inform. (2022) 26:1432–40. doi: 10.1109/JBHI.2021.3083187, PMID: 34029201

[14] Nensa F Demircioglu A Rischpler C. Artificial intelligence in nuclear medicine. J Nucl Med. (2019) 60:29S–37S. doi: 10.2967/jnumed.118.220590, PMID: 31481587

[15] Lecun Y Bengio Y. Convolutional Networks for Images, Speech, and Time-Series. Handbook of Brain Theory & Neural Networks. Computer Science, Linguistics (1998).

[16] Berger AC Korkut A Kanchi RS Hegde AM Lenoir W Liu W. A comprehensive pan-cancer molecular study of gynecologic and breast cancers. Cancer Cell. (2018) 33:690–705.e9. doi: 10.1016/j.ccell.2018.03.014, PMID: 29622464 PMC5959730

[17] Hosny A Parmar C Quackenbush J Schwartz LH Aerts HJWL. Artificial intelligence in radiology. Nat Rev Cancer. (2018) 18:500–10. doi: 10.1038/s41568-018-0016-5, PMID: 29777175 PMC6268174

[18] Hricak H Abdel-Wahab M Atun R Lette MM Paez D Brink JA. Medical imaging and nuclear medicine: a Lancet Oncology Commission. Lancet Oncol. (2021) 22:e136–72. doi: 10.1016/S1470-2045(20)30751-8, PMID: 33676609 PMC8444235

[19] Hart GR Roffman DA Decker R Deng J. A multi-parameterized artificial neural network for lung cancer risk prediction. PloS One. (2018) 13:e0205264. doi: 10.1371/journal.pone.0205264, PMID: 30356283 PMC6200229

[20] Matsubara T Miyatake Y Yaguchi T. The symplectic adjoint method: memory-efficient backpropagation of neural-network-based differential equations. IEEE Trans Neural Netw Learn Syst. (2024) 35:10526–38. doi: 10.1109/TNNLS.2023.3242345, PMID: 37027779

[21] Unger M Kather JN. Deep learning in cancer genomics and histopathology. Genome Med. (2024) 16:44. doi: 10.1186/s13073-024-01315-6, PMID: 38539231 PMC10976780

[22] Shmatko A Ghaffari Laleh N Gerstung M Kather JN. Artificial intelligence in histopathology: enhancing cancer research and clinical oncology. Nat Cancer. (2022) 3:1026–38. doi: 10.1038/s43018-022-00436-4, PMID: 36138135

[23] Alsalatie M Alquran H Mustafa WA Zyout A Alqudah AM Kaifi R. A new weighted deep learning feature using particle swarm and ant lion optimization for cervical cancer diagnosis on pap smear images. Diagnostics (Basel). (2023) 13:2762. doi: 10.3390/diagnostics13172762, PMID: 37685299 PMC10487265

[24] Ghosh D Cabrera J. Enriched random forest for high dimensional genomic data. IEEE/ACM Trans Comput Biol Bioinform. (2022) 19:2817–28. doi: 10.1109/TCBB.2021.3089417, PMID: 34129502 PMC9923687

[25] Pfeifer B Holzinger A Schimek MG. Robust random forest-based all-relevant feature ranks for trustworthy AI. Stud Health Technol Inform. (2022) 294:137–8. doi: 10.3233/SHTI220418, PMID: 35612038

[26] Fotouhi S Asadi S Kattan MW. A comprehensive data level analysis for cancer diagnosis on imbalanced data. J BioMed Inform. (2019) 90:103089. doi: 10.1016/j.jbi.2018.12.003, PMID: 30611011

[27] Wlodzislaw D Biesiada J Winiarski T Grabczewski K. Feature selection based on information theory filters and feature elimination wrapper methods. (2022). Available at: https://www.researchgate.net/publication/239200508.

[28] Theodoridis S Koutroumbas K. Clustering Algorithms II: Hierarchical Algorithms. In: Pattern Recognition, 4th ed. (2009). p. 653–700.

[29] Pergialiotis V Pouliakis A Parthenis C Damaskou V Chrelias C Papantoniou N. The utility of artificial neural networks and classification and regression trees for the prediction of endometrial cancer in postmenopausal women. Public Health. (2018) 164:1–6. doi: 10.1016/j.puhe.2018.07.012, PMID: 30149185

[30] Yao Z Ruzzo WL. A regression-based K nearest neighbor algorithm for gene function prediction from heterogeneous data. BMC Bioinf. (2006) Suppl 1:S11. doi: 10.1186/1471-2105-7-S1-S11, PMID: 16723004 PMC1810312

[31] Yan Y Yao XJ Wang SH Zhang YD. A survey of computer-aided tumor diagnosis based on convolutional neural network. Biol (Basel). (2021) 10:1084. doi: 10.3390/biology10111084, PMID: 34827077 PMC8615026

[32] Kourou K Exarchos TP Exarchos KP Karamouzis MV Fotiadis DI. Machine learning applications in cancer prognosis and prediction. Comput Struct Biotechnol J. (2014) 13:8–17. doi: 10.1016/j.csbj.2014.11.005, PMID: 25750696 PMC4348437

[33] Vidya R Nasira GM. Prediction of cervical cancer using hybrid induction technique: A solution for human hereditary disease patterns. Indian J Sci Technol. (2016) 9. doi: 10.17485/ijst/2016/v9i30/82085

[34] Chang CC Cheng SL Lu CJ Liao KH. Prediction of recurrence in patients with cervical cancer using MARS and classification. Int J Mach Learn computing. (2013) 3:75:8. doi: 10.7763/IJMLC.2013.V3.276

[35] Soumya K Sneha K Arunvinodh C. Cervical cancer detection and classification using texture analysis. Biomed Pharmacol J. (2016) 9:663–71. doi: 10.13005/bpj/988

[36] Vijayan A Fatima S Sowmya A Vafaee F. Blood-based transcriptomic signature panel identification for cancer diagnosis: benchmarking of feature extraction methods. Brief Bioinform. (2022) 23:bbac315. doi: 10.1093/bib/bbac315, PMID: 35945147

[37] Koch FC Sutton GJ Voineagu I Vafaee F. Supervised application of internal validation measures to benchmark dimensionality reduction methods in scRNA-seq data. Brief Bioinform. (2021) 22:bbab304. doi: 10.1093/bib/bbab304, PMID: 34374742

[38] Chen H Lu Y Yang Y Rao Y. A drug combination prediction framework based on graph convolutional network and heterogeneous information. IEEE/ACM Trans Comput Biol Bioinform. (2023) 20:1917–25. doi: 10.1109/TCBB.2022.3224734, PMID: 36427284

[39] Gan Y Huang X Guo W Yan C Zou G. Predicting synergistic anticancer drug combination based on low-rank global attention mechanism and bilinear predictor. Bioinformatics. (2023) 39:btad607. doi: 10.1093/bioinformatics/btad607, PMID: 37812255 PMC10598574

[40] Li W Dong S Wang H Wu R Wu H Tang ZR. Risk analysis of pulmonary metastasis of chondrosarcoma by establishing and validating a new clinical prediction model: a clinical study based on SEER database. BMC Musculoskelet Disord. (2021) 22:529. doi: 10.1186/s12891-021-04414-2, PMID: 34107945 PMC8191035

[41] Guo S Tian M Fan Y Zhang X. Recent advances in mass spectrometry-based proteomics and metabolomics in chronic rhinosinusitis with nasal polyps. Front Immunol. (2023) 14:1267194. doi: 10.3389/fimmu.2023.1267194, PMID: 37744372 PMC10511644

[42] Chen RJ Lu MY Williamson DFK Chen TY Lipkova J Noor Z. Pan-cancer integrative histology-genomic analysis via multimodal deep learning. Cancer Cell. (2022) 40:865–878.e6. doi: 10.1016/j.ccell.2022.07.004, PMID: 35944502 PMC10397370

[43] Aljuaid H Alturki N Alsubaie N Cavallaro L Liotta A. Computer-aided diagnosis for breast cancer classification using deep neural networks and transfer learning. Comput Methods Programs Biomed. (2022) 223:106951. doi: 10.1016/j.cmpb.2022.106951, PMID: 35767911

[44] Hamidinekoo A Suhail Z Qaiser T Zwiggelaar R. Investigating the Effect of Various Augmentations on the Input Data Fed to a Convolutional Neural Network for the Task of Mammographic Mass Classification[C]//Annual Conference on Medical Image Understanding and Analysis. (2017). doi: 10.1007/978-3-319-60964-5_35

[45] Schmidhuber J. Deep learning in neural networks: an overview. Neural Netw. (2015) 61:85–117. doi: 10.1016/j.neunet.2014.09.003, PMID: 25462637

[46] Arun Kumar S Sasikala S. Review on deep learning-based CAD systems for breast cancer diagnosis. Technol Cancer Res Treat. (2023) 22:15330338231177977. doi: 10.1177/15330338231177977, PMID: 37282580 PMC10272643

[47] Lei R Yu Y Li Q Yao Q Wang J Gao M. Deep learning magnetic resonance imaging predicts platinum sensitivity in patients with epithelial ovarian cancer. Front Oncol. (2022) 12:895177. doi: 10.3389/fonc.2022.895177, PMID: 36505880 PMC9727155

[48] Yao G Lei T Zhong J. A review of convolutional-neural-network-based action recognition. Pattern Recognition Lett. (2018) 118:14–22. doi: 10.1016/j.patrec.2018.05.018

[49] Yamashita R Nishio M Do RKG Togashi K. Convolutional neural networks: an overview and application in radiology. Insights Imaging. (2018) 9:611–29. doi: 10.1007/s13244-018-0639-9, PMID: 29934920 PMC6108980

[50] Nirthika R Manivannan S Ramanan A Wang R. Pooling in convolutional neural networks for medical image analysis: a survey and an empirical study. Neural Comput Appl. (2022) 34:5321–47. doi: 10.1007/s00521-022-06953-8, PMID: 35125669 PMC8804673

[51] Shrestha P Poudyal B Yadollahi S E Wright D V Gregory A D Warner J. A systematic review on the use of artificial intelligence in gynecologic imaging - Background, state of the art, and future directions. Gynecol Oncol. (2022) 166:596–605. doi: 10.1016/j.ygyno.2022.07.024, PMID: 35914978

[52] Albawi S Mohammed TA Alzawi S. Understanding of a Convolutional Neural Network[J]. (2017). doi: 10.1109/ICEngTechnol.2017.8308186

[53] Sun M Song Z Jiang X Pan J Pang Y. Learning pooling for convolutional neural network. Neurocomputing. (2017) 224:96–104. doi: 10.1016/j.neucom.2016.10.049

[54] Xu F Zhang X Xin Z Yang A. Investigation on the chinese text sentiment analysis based on convolutional neural networks in deep learning. Computers Materials Continua. (2019) 58:697–709. doi: 10.32604/cmc.2019.05375

[55] Ghoniem RM Algarni AD Refky B Ewees AA. Multi-modal evolutionary deep learning model for ovarian cancer diagnosis. Symmetry. (2021) 4). doi: 10.3390/SYM13040643

[56] Krizhevsky A Sutskever I Hinton G. (2012). ImageNet Classification with Deep Convolutional Neural Networks[C]//NIPS.Curran Associates Inc. doi: 10.1145/3065386

[57] Szegedy C Liu W Jia Y Sermanet P Reed S Anguelov D. Going Deeper with Convolutions[J]. IEEE Computer Society (2014). doi: 10.1109/CVPR.2015.7298594

[58] Zhang W Li R Deng H Sken D. Deep convolutional neural networks for multi-modality isointense infant brain image segmentation. NeuroImage. (2015) 108:214–24. doi: 10.1016/j.neuroimage.2014.12.061, PMID: 25562829 PMC4323729

[59] Sato M Horie K Hara A Miyamoto Y Kurihara K Tomio K. Application of deep learning to the classification of images from colposcopy. Oncol Lett. (2018) 15:3518–23. doi: 10.3892/ol.2018.7762, PMID: 29456725 PMC5795879

[60] He K Zhang X Ren S Sun J. Deep Residual Learning for Image Recognition. IEEE, Conference: Introduction to Deep Neural Networks At: PIEAS, Islamabad, Pakistan (2016). doi: 10.1109/CVPR.2016.90

[61] Ronneberger O Fischer P Brox T. U-Net: Convolutional Networks for Biomedical Image Segmentation[J], Springer, Cham (2015). doi: 10.1007/978-3-662-54345-0_3

[62] Zhang D Yang Z Jiang S Zhou Z Meng M Wang W. Automatic segmentation and applicator reconstruction for CT-based brachytherapy of cervical cancer using 3D convolutional neural networks. J Appl Clin Med Phys. (2020) 21:158–69. doi: 10.1002/acm2.13024, PMID: 32991783 PMC7592978

[63] Goodfellow IJ Pouget-Abadie J Mirza M Warde-Farley D Ozair S Courville A. Generative Adversarial Networks. MIT Press (2014) 2:2672–268. doi: 10.3156/JSOFT.29.5_177_2

[64] Levine AB Peng J Farnell D Nursey M Wang Y Naso JR. Synthesis of diagnostic quality cancer pathology images by generative adversarial networks. J Pathol. (2020) 252:178–88. doi: 10.1002/path.5509, PMID: 32686118

[65] Gu J Wang Z Kuen J Ma L Gang W. Recent advances in convolutional neural networks. Pattern Recognition. (2018) 77:354–77. doi: 10.1016/j.patcog.2017.10.013

[66] Chen H Zhang Y Zhang W Liao P Li K Zhou J. Low-dose CT via convolutional neural network. BioMed Opt Express. (2017) 8:679–94. doi: 10.1364/BOE.8.000679, PMID: 28270976 PMC5330597

[67] Dosovitskiy A Beyer L Kolesnikov A Weissenborn D Houlsby N. An Image is Worth 16x16 Words: Transformers for Image Recognition at Scale[J]. (2020). doi: 10.48550/arXiv.2010.11929

[68] Jie Z Ganqu C Shengding H Liu J Pan L Xiong H. Graph neural networks: A review of methods and applications. AI Open. (2020) 1:57–81. doi: 10.48550/arXiv.2202.13852

[69] Besharatifard M Vafaee F. A review on graph neural networks for predicting synergistic drug combinations. Artif Intell Revie. (2024) 57:38. doi: 10.1007/s10462-023-10669-z

[70] Shao K Zhang Y Wen Y Zhang Z He S Bo X. DTI-HETA: prediction of drug-target interactions based on GCN and GAT on heterogeneous graph. Brief Bioinform. (2022) 23:bbac109. doi: 10.1093/bib/bbac109, PMID: 35380622

[71] Lin M Wen K Zhu X Zhao H Sun X. Graph autoencoder with preserving node attribute similarity. Entropy (Basel). (2023) 25:567. doi: 10.3390/e25040567, PMID: 37190356 PMC10138145

[72] Hamilton WL Ying R Leskovec J. Inductive representation learning on large graphs. (2017), 1025–10. doi: 10.48550/arXiv.1706.02216

[73] Hansen L Heinrich MP. GraphRegNet: deep graph regularisation networks on sparse keypoints for dense registration of 3D lung CTs. IEEE Trans Med Imaging. (2021) 40:2246–57. doi: 10.1109/TMI.2021.3073986, PMID: 33872144

[74] Kodipalli A Fernandes SL Dasar S. An empirical evaluation of a novel ensemble deep neural network model and explainable AI for accurate segmentation and classification of ovarian tumors using CT images. Diagnostics (Basel). (2024) 14:543. doi: 10.3390/diagnostics14050543, PMID: 38473015 PMC10930928

[75] Zhou X Wang H Feng C Xu R He Y Li L. Emerging applications of deep learning in bone tumors: current advances and challenges. Front Oncol. (2022) 12:908873. doi: 10.3389/fonc.2022.908873, PMID: 35928860 PMC9345628

[76] Towner M Kim JJ Simon MA Matei D Roque D. Disparities in gynecologic cancer incidence, treatment, and survival: a narrative review of outcomes among black and white women in the United States. Int J Gynecol Cancer. (2022) 32:931–8. doi: 10.1136/ijgc-2022-003476, PMID: 35523443 PMC9509411

[77] Bray F Laversanne M Sung H Ferlay J Siegel RL Soerjomataram I. Global cancer statistics 2022: GLOBOCAN estimates of incidence and mortality worldwide for 36 cancers in 185 countries. CA Cancer J Clin. (2024) 74:229–63. doi: 10.3322/caac.21834, PMID: 38572751

[78] Siegel RL Miller KD Fuchs HE Jemal A. Cancer statistics, 2022. CA Cancer J Clin. (2022) 72:7–33. doi: 10.3322/caac.21708, PMID: 35020204

[79] Gadducci A Cosio S. Screening for ovarian cancer in the general population: state of art and perspectives of clinical research. Anticancer Res. (2022) 42:4207–16. doi: 10.21873/anticanres.15921, PMID: 36039417

[80] Bignardi T Condous G. Ultrasound for ovarian cancer screening: are we throwing the baby out with the bath water? Gynecol Obstet Invest. (2011) 71:41–6. doi: 10.1159/000320731, PMID: 21160193

[81] Montagnana M Benati M Danese E. Circulating biomarkers in epithelial ovarian cancer diagnosis: from present to future perspective. Ann Transl Med. (2017) 5:276. doi: 10.21037/atm.2017.05.13, PMID: 28758102 PMC5515813

[82] Veneziani AC Gonzalez-Ochoa E Alqaisi H Madariaga A Bhat G Rouzbahman M. Heterogeneity and treatment landscape of ovarian carcinoma. Nat Rev Clin Oncol. (2023) 20:820–42. doi: 10.1038/s41571-023-00819-1, PMID: 37783747

[83] Ding H Hu B Guo R. Comprehensive analysis of single cell and bulk data develops a promising prognostic signature for improving immunotherapy responses in ovarian cancer. PloS One. (2024) 19:e0298125. doi: 10.1371/journal.pone.0298125, PMID: 38346070 PMC10861092

[84] Colombo N Sessa C du Bois A Ledermann J McCluggage WG McNeish I. ESMO-ESGO consensus conference recommendations on ovarian cancer: pathology and molecular biology, early and advanced stages, borderline tumours and recurrent disease†. Ann Oncol. (2019) 30:672–705. doi: 10.1093/annonc/mdz062, PMID: 31046081

[85] Zhao H Sun Q Li L Zhou J Zhang C Hu T. High expression levels of AGGF1 and MFAP4 predict primary platinum-based chemoresistance and are associated with adverse prognosis in patients with serous ovarian cancer. J Cancer. (2019) 10:397–407. doi: 10.7150/jca.28127, PMID: 30719133 PMC6360311

[86] Edaily S Abdel-Razeq H. Management strategies of breast cancer patients with *BRCA1* and *BRCA2* pathogenic germline variants. Onco Targets Ther. (2022) 15:815–26. doi: 10.2147/OTT.S369844, PMID: 35923470 PMC9343017

[87] Kruczkowski M Drabik-Kruczkowska A Marciniak A Tarczewska M Kosowska M Szczerska M. Predictions of cervical cancer identification by photonic method combined with machine learning. Sci Rep. (2022) 12:3762. doi: 10.1038/s41598-022-07723-1, PMID: 35260666 PMC8904553

[88] Pedersen K Fogelberg S Thamsborg LH Clements M Nygård M Kristiansen IS. An overview of cervical cancer epidemiology and prevention in Scandinavia. Acta Obstet Gynecol Scand. (2018) 97:795–807. doi: 10.1111/aogs.13313, PMID: 29388202

[89] Tamura K Hasegawa K Katsumata N Matsumoto K Mukai H Takahashi S. Efficacy and safety of nivolumab in Japanese patients with uterine cervical cancer, uterine corpus cancer, or soft tissue sarcoma: Multicenter, open-label phase 2 trial. Cancer Sci. (2019) 110:2894–904. doi: 10.1111/cas.14148, PMID: 31348579 PMC6726684

[90] Hong DS Concin N Vergote I de Bono JS Slomovitz BM Drew Y. Tisotumab vedotin in previously treated recurrent or metastatic cervical cancer. Clin Cancer Res. (2020) 26:1220–8. doi: 10.1158/1078-0432.CCR-19-2962, PMID: 31796521

[91] Li J Lai H Qin H Zhou D Zhao Y Sheng X. Current status of high-risk HPV infection and correlation with multiple infections in cervical lesions in Western Guangzhou. Front Med (Lausanne). (2024) 11:1252073. doi: 10.3389/fmed.2024.1252073, PMID: 38695017 PMC11061398

[92] Zhang Y Du H Wang C Huang X Qu X Wu R. Feasibility and applicability of self-sampling based online cervical cancer screening: findings from the China online cervical cancer screening trial. Infect Agent Cancer. (2024) 19:16. doi: 10.1186/s13027-024-00583-6, PMID: 38664748 PMC11046965

[93] Minnaar CA Kotzen JA Ayeni OA Naidoo T Tunmer M Sharma V. The effect of modulated electro-hyperthermia on local disease control in HIV-positive and -negative cervical cancer women in South Africa: Early results from a phase III randomised controlled trial. PloS One. (2019) 14:e0217894. doi: 10.1371/journal.pone.0217894, PMID: 31216321 PMC6584021

[94] Castle PE Kinney WK Cheung LC Gage JC Fetterman B Poitras NE. Why does cervical cancer occur in a state-of-the-art screening program? Gynecol Oncol. (2017) 146:546–53. doi: 10.1016/j.ygyno.2017.06.003, PMID: 28606721 PMC5743197

[95] Luo H Du H Belinson JL Wu R. Evaluation of alternately combining HPV viral load and 16/18 genotyping in secondary screening algorithms. PloS One. (2019) 14:e0220200. doi: 10.1371/journal.pone.0220200, PMID: 31348794 PMC6660090

[96] Sadeghi F Mostaghimi T Taheri M Yazdani S Javadian M Ranaee M. Investigating the role of Epstein-Barr virus and human papillomavirus types 16 and 18 co-infections in cervical disease of Iranian women. Front Oncol. (2024) 14:1331862. doi: 10.3389/fonc.2024.1331862, PMID: 38720799 PMC11076674

[97] Duan Z Xu C Li Z Feng B Nie C. FMA-net: fusion of multi-scale attention for grading cervical precancerous lesions. Mathematics. (2024) 12:2227–7390. doi: 10.3390/math12070958

[98] Sung H Ferlay J Siegel RL Laversanne M Soerjomataram I Jemal A. Global cancer statistics 2020: GLOBOCAN estimates of incidence and mortality worldwide for 36 cancers in 185 countries. CA Cancer J Clin. (2021) 71:209–49. doi: 10.3322/caac.21660, PMID: 33538338

[99] Makker V MacKay H Ray-Coquard I Levine DA Westin SN Aoki D. Endometrial cancer. Nat Rev Dis Primers. (2021) 7:88. doi: 10.1038/s41572-021-00324-8, PMID: 34887451 PMC9421940

[100] Nguyen PN Nguyen VT. Evaluating clinical features in intracavitary uterine pathologies among Vietnamese women presenting with peri-and postmenopausal bleeding: A bicentric observational descriptive analysis. J Midlife Health. (2022) 13:225–32. doi: 10.4103/jmh.jmh_81_22, PMID: 36950211 PMC10025815

[101] Schramm A Ebner F Bauer E Janni W Friebe-Hoffmann U Pellegrino M. Value of endometrial thickness assessed by transvaginal ultrasound for the prediction of endometrial cancer in patients with postmenopausal bleeding. Arch Gynecol Obstet. (2017) 296:319–26. doi: 10.1007/s00404-017-4439-0, PMID: 28634754

[102] Lax SF. Pathology of endometrial carcinoma. Adv Exp Med Biol. (2017) 943:75–96. doi: 10.1007/978-3-319-43139-0_3, PMID: 27910065

[103] Lu Z Chen J. Introduction of WHO classification of tumours of female reproductive organs, fourth edition. Zhonghua bing li xue za zhi Chin J Pathol. (2014) 43:649–50. doi: 10.3760/cma.j.issa0529-5807.2014.10.001, PMID: 25567588

[104] Concin N Matias-Guiu X Vergote I Cibula D Mirza MR Marnitz S. ESGO/ESTRO/ESP guidelines for the management of patients with endometrial carcinoma. Radiother Oncol. (2021) 154:327–53. doi: 10.1016/j.radonc.2020.11.018, PMID: 33712263

[105] Berek JS Matias-Guiu X Creutzberg C Fotopoulou C Gaffney D Kehoe S. Endometrial Cancer Staging Subcommittee, FIGO Women’s Cancer Committee. FIGO staging of endometrial cancer: 2023. J Gynecol Oncol. (2023) 34:e85. doi: 10.3802/jgo.2023.34.e85, PMID: 37593813 PMC10482588

[106] Xu X Desai VB Schwartz PE Gross CP Lin H Schymura MJ. Safety warning about laparoscopic power morcellation in hysterectomy: A cost-effectiveness analysis of national impact. Womens Health Rep (New Rochelle). (2022) 3:369–84. doi: 10.1089/whr.2021.0101, PMID: 35415718 PMC8994439

[107] Sachdev JC Sandoval AC Jahanzeb M. Update on precision medicine in breast cancer. Cancer Treat Res. (2019) 178:45–80. doi: 10.1007/978-3-030-16391-4_2, PMID: 31209841

[108] Azuaje F. Artificial intelligence for precision oncology: beyond patient stratification. NPJ Precis Oncol. (2019) 3:6. doi: 10.1038/s41698-019-0078-1, PMID: 30820462 PMC6389974

[109] Harvey C Koubek R Bégat V Jacob S. Usability evaluation of a blood glucose monitoring system with a spill-resistant vial, easier strip handling, and connectivity to a mobile app: improvement of patient convenience and satisfaction. J Diabetes Sci Technol. (2016) 10:1136–41. doi: 10.1177/1932296816658058, PMID: 27390222 PMC5032967

[110] Lambin P Rios-Velazquez E Leijenaar R Carvalho S van Stiphout RG Granton P. Radiomics: extracting more information from medical images using advanced feature analysis. Eur J Cancer. (2012) 48:441–6. doi: 10.1016/j.ejca.2011.11.036, PMID: 22257792 PMC4533986

[111] Tagliafico AS Piana M Schenone D Lai R Massone AM Houssami N. Overview of radiomics in breast cancer diagnosis and prognostication. Breast. (2020) 49:74–80. doi: 10.1016/j.breast.2019.10.018, PMID: 31739125 PMC7375670

[112] Thomas HMT Wang HYC Varghese AJ Donovan EM South CP Saxby H. Reproducibility in radiomics: A comparison of feature extraction methods and two independent datasets. Appl Sci (Basel). (2024) 166. doi: 10.3390/app13127291, PMID: 38725869 PMC7615943

[113] Savadjiev P Chong J Dohan A Agnus V Forghani R Reinhold C. Image-based biomarkers for solid tumor quantification. Eur Radiol. (2019) 29:5431–40. doi: 10.1007/s00330-019-06169-w, PMID: 30963275

[114] Lambin P Leijenaar RTH Deist TM Peerlings J de Jong EEC van Timmeren J. Radiomics: the bridge between medical imaging and personalized medicine. Nat Rev Clin Oncol. (2017) 14:749–62. doi: 10.1038/nrclinonc.2017.141, PMID: 28975929

[115] Sun R Limkin EJ Vakalopoulou M Dercle L Champiat S Han SR. A radiomics approach to assess tumour-infiltrating CD8 cells and response to anti-PD-1 or anti-PD-L1 immunotherapy: an imaging biomarker, retrospective multicohort study. Lancet Oncol. (2018) 19:1180–91. doi: 10.1016/S1470-2045(18)30413-3, PMID: 30120041

[116] Huang S Yang J Fong S Zhao Q. Artificial intelligence in cancer diagnosis and prognosis: Opportunities and challenges. Cancer Lett. (2020) 471:61–71. doi: 10.1016/j.canlet.2019.12.007, PMID: 31830558

[117] Eweje FR Bao B Wu J Dalal D Liao WH He Y. Deep learning for classification of bone lesions on routine MRI. EBioMedicine. (2021) 68:103402. doi: 10.1016/j.ebiom.2021.103402, PMID: 34098339 PMC8190437

[118] Chen J Chen Y Sun K Wang Y He H Sun L. Prediction of ovarian cancer-related metabolites based on graph neural network. Front Cell Dev Biol. (2021) 9:753221. doi: 10.3389/fcell.2021.753221, PMID: 34676219 PMC8525679

[119] Schwartz D Sawyer TW Thurston N Barton J Ditzler G. Ovarian cancer detection using optical coherence tomography and convolutional neural networks. Neural Comput Appl. (2022) 34:8977–87. doi: 10.1007/s00521-022-06920-3, PMID: 35095211 PMC8785933

[120] Tanabe K Ikeda M Hayashi M Matsuo K Yasaka M Machida H. Comprehensive serum glycopeptide spectra analysis combined with artificial intelligence (CSGSA-AI) to diagnose early-stage ovarian cancer. Cancers (Basel). (2020) 12:2373. doi: 10.3390/cancers12092373, PMID: 32825730 PMC7563497

[121] Elakkiya R Teja KSS Deborah LJ Bisogni C Medaglia C. Imaging based cervical cancer diagnostics using small object detection - generative adversarial networks. Multimedia Tools Appl. (2022) 81:191. doi: 10.1007/s11042-021-10627-3

[122] Fekri-Ershad S Alsaffar MF. Developing a tuned three-layer perceptron fed with trained deep convolutional neural networks for cervical cancer diagnosis. Diagnostics (Basel). (2023) 13:686. doi: 10.3390/diagnostics13040686, PMID: 36832174 PMC9955324

[123] Chandran V Sumithra MG Karthick A George T Deivakani M Elakkiya B. Diagnosis of Cervical Cancer based on Ensemble Deep Learning Network using Colposcopy Images. BioMed Res Int. (2021) 2021:5584004. doi: 10.1155/2021/5584004, PMID: 33997017 PMC8112909

[124] Takahashi Y Sone K Noda K Yoshida K Toyohara Y Kato K. Automated system for diagnosing endometrial cancer by adopting deep-learning technology in hysteroscopy. PloS One. (2021) 16:e0248526. doi: 10.1371/journal.pone.0248526, PMID: 33788887 PMC8011803

[125] Qin X Hu X Xiao W Zhu C Ma Q Zhang C. Preoperative evaluation of hepatocellular carcinoma differentiation using contrast-enhanced ultrasound-based deep-learning radiomics model. J Hepatocell Carcinoma. (2023) 10:157–68. doi: 10.2147/JHC.S400166, PMID: 36789250 PMC9922506

[126] Tan SL Selvachandran G Ding W Paramesran R Kotecha K. Cervical cancer classification from pap smear images using deep convolutional neural network models. Interdiscip Sci. (2024) 16:16–38. doi: 10.1007/s12539-023-00589-5, PMID: 37962777 PMC10881721

[127] Wen B Campbell KR Tilbury K Nadiarnykh O Brewer MA Patankar M. 3D texture analysis for classification of second harmonic generation images of human ovarian cancer. Sci Rep. (2016) 6:35734. doi: 10.1038/srep35734, PMID: 27767180 PMC5073303

[128] Wu M Yan C Liu H Liu Q. Automatic classification of ovarian cancer types from cytological images using deep convolutional neural networks. Biosci Rep. (2018) 38:BSR20180289. doi: 10.1042/BSR20180289, PMID: 29572387 PMC5938423

[129] Liu S Yuan Z Qiao X Liu Q Song K Kong B. Light scattering pattern specific convolutional network static cytometry for label-free classification of cervical cells. Cytometry A. (2021) 99:610–21. doi: 10.1002/cyto.a.24349, PMID: 33840152

[130] Li Q Wang R Xie Z Zhao L Wang Y Sun C. Clinically applicable pathological diagnosis system for cell clumps in endometrial cancer screening via deep convolutional neural networks. Cancers (Basel). (2022) 14:4109. doi: 10.3390/cancers14174109, PMID: 36077646 PMC9454725

[131] Yang J Cao Y Zhou F Li C Lv J Li P. Combined deep-learning MRI-based radiomic models for preoperative risk classification of endometrial endometrioid adenocarcinoma. Front Oncol. (2023) 13:1231497. doi: 10.3389/fonc.2023.1231497, PMID: 37909025 PMC10613647

[132] Bell LC Shimron E. Sharing data is essential for the future of AI in medical imaging. Radiol Artif Intell. (2024) 6:e230337. doi: 10.1148/ryai.230337, PMID: 38231036 PMC10831510

[133] Kodipalli A Fernandes SL Gururaj V Varada Rameshbabu S Dasar S. Performance analysis of segmentation and classification of CT-scanned ovarian tumours using U-net and deep convolutional neural networks. Diagnostics (Basel). (2023) 13:2282. doi: 10.3390/diagnostics13132282, PMID: 37443676 PMC10341135

[134] Kudva V Prasad K Guruvare S. Automation of detection of cervical cancer using convolutional neural networks. Crit Rev BioMed Eng. (2018) 46:135–45. doi: 10.1615/CritRevBiomedEng.2018026019, PMID: 30055530

[135] Hodneland E Dybvik JA Wagner-Larsen KS Šoltészová V Munthe-Kaas AZ Fasmer KE. Automated segmentation of endometrial cancer on MR images using deep learning. Sci Rep. (2021) 11:179. doi: 10.1038/s41598-020-80068-9, PMID: 33420205 PMC7794479

[136] Wang N Khan S Elo LL. VarSCAT: A computational tool for sequence context annotations of genomic variants. PloS Comput Biol. (2023) 19:e1010727. doi: 10.1371/journal.pcbi.1010727, PMID: 37566612 PMC10446208

[137] Guan P Sung WK. Structural variation detection using next-generation sequencing data: A comparative technical review. Methods. (2016) 102:36–49. doi: 10.1016/j.ymeth.2016.01.020, PMID: 26845461

[138] Yang L Yin H Bai L Yao W Tao T Zhao Q. Mapping and functional characterization of structural variation in 1060 pig genomes. Genome Biol. (2024) 25:116. doi: 10.1186/s13059-024-03253-3, PMID: 38715020 PMC11075355

[139] Tutt A Tovey H Cheang MCU Kernaghan S Kilburn L Gazinska P. Carboplatin in BRCA1/2-mutated and triple-negative breast cancer BRCAness subgroups: the TNT Trial. Nat Med. (2018) 24:628–37. doi: 10.1038/s41591-018-0009-7, PMID: 29713086 PMC6372067

[140] Moore K Colombo N Scambia G Kim BG Oaknin A Friedlander M. Maintenance olaparib in patients with newly diagnosed advanced ovarian cancer. N Engl J Med. (2018) 379:2495–505. doi: 10.1056/NEJMoa1810858, PMID: 30345884

[141] Hu J Szymczak S. Evaluation of network-guided random forest for disease gene discovery. BioData Min. (2024) 17:10. doi: 10.1186/s13040-024-00361-5, PMID: 38627770 PMC11020917

[142] Bahado-Singh RO Ibrahim A Al-Wahab Z Aydas B Radhakrishna U Yilmaz A. Precision gynecologic oncology: circulating cell free DNA epigenomic analysis, artificial intelligence and the accurate detection of ovarian cancer. Sci Rep. (2022) 12:18625. doi: 10.1038/s41598-022-23149-1, PMID: 36329159 PMC9633647

[143] Guo ZH Chen ZH You ZH Wang YB Yi HC Wang MN. A learning-based method to predict LncRNA-disease associations by combining CNN and ELM. BMC Bioinf. (2022) 22:622. doi: 10.1186/s12859-022-04611-3, PMID: 35317723 PMC8941737

[144] Manganaro L Lakhman Y Bharwani N Gui B Gigli S Vinci V. Staging, recurrence and follow-up of uterine cervical cancer using MRI: Updated Guidelines of the European Society of Urogenital Radiology after revised FIGO staging 2018. Eur Radiol. (2021) 31:7802–16. doi: 10.1007/s00330-020-07632-9, PMID: 33852049

[145] Tangjitgamol S Manusirivithaya S Jesadapatarakul S Leelahakorn S Thawaramara T. Lymph node size in uterine cancer: A revisit. Int J Gynecol Cancer. (2006) 16:1880–4. doi: 10.1111/j.1525-1438.2006.00715.x, PMID: 17009986

[146] Napel S Mu W Jardim-Perassi BV Aerts HJWL Gillies RJ. Quantitative imaging of cancer in the postgenomic era: Radio(geno)mics, deep learning, and habitats. Cancer. (2018) 124:4633–49. doi: 10.1002/cncr.31630, PMID: 30383900 PMC6482447

[147] Liu Y Fan H Dong D Liu P He B Meng L. Computed tomography-based radiomic model at node level for the prediction of normal-sized lymph node metastasis in cervical cancer. Transl Oncol. (2021) 14:101113. doi: 10.1016/j.tranon.2021.101113, PMID: 33975178 PMC8131712

[148] Urase Y Nishio M Ueno Y Kono AK Murakami T. Simulation study of low-dose sparse-sampling CT with deep learning-based reconstruction: usefulness for evaluation of ovarian cancer metastasis. Appl Sci. (2020) 10:4446. doi: 10.3390/app10134446

[149] Qian W Li Z Chen W Yin H Zhang J Xu J. RESOLVE-DWI-based deep learning nomogram for prediction of normal-sized lymph node metastasis in cervical cancer: a preliminary study. BMC Med Imaging. (2022) 22:221. doi: 10.1186/s12880-022-00948-6, PMID: 36528577 PMC9759891

[150] Feng M Zhao Y Chen J Zhao T Mei J Fan Y. A deep learning model for lymph node metastasis prediction based on digital histopathological images of primary endometrial cancer. Quant Imaging Med Surg. (2023) 13:1899–913. doi: 10.21037/qims-22-220, PMID: 36915334 PMC10006118

[151] Bois AD Sehouli J Vergote I Ferron G Harter P. Randomized phase III study to evaluate the impact of secondary cytoreductive surgery in recurrent ovarian cancer: Final analysis of AGO DESKTOP III/ENGOT-ov20. J Clin Oncol. (2020) 38:6000–0. doi: 10.1200/JCO.2020.38.15_suppl.6000

[152] Cook SA Tinker AV. PARP inhibitors and the evolving landscape of ovarian cancer management: A review. BioDrugs. (2019) 33:255–73. doi: 10.1007/s40259-019-00347-4, PMID: 30895466

[153] Yu KH Zhang C Berry GJ Altman RB Ré C Rubin DL. Predicting non-small cell lung cancer prognosis by fully automated microscopic pathology image features. Nat Commun. (2016) 7:12474. doi: 10.1038/ncomms12474, PMID: 27527408 PMC4990706

[154] Zhuang H Li B Ma J Monkam P Qi S Qian W. An attention-based deep learning network for predicting platinum resistance in ovarian cancer. IEEE Access. (2024) 12:153273. doi: 10.1109/ACCESS.2024.3377560

[155] Lao J Chen Y Li ZC Li Q Zhang J Liu J. A deep learning-based radiomics model for prediction of survival in glioblastoma multiforme. Sci Rep. (2017) 7:10353. doi: 10.1038/s41598-017-10649-8, PMID: 28871110 PMC5583361

[156] Hötker AM Tarlinton L Mazaheri Y Woo KM Gönen M Saltz LB. Multiparametric MRI in the assessment of response of rectal cancer to neoadjuvant chemoradiotherapy: A comparison of morphological, volumetric and functional MRI parameters. Eur Radiol. (2016) 26:4303–12. doi: 10.1007/s00330-016-4283-9, PMID: 26945761 PMC5203699

[157] Gillies RJ Kinahan PE Hricak H. Radiomics: images are more than pictures, they are data. Radiology. (2016) 278:563–77. doi: 10.1148/radiol.2015151169, PMID: 26579733 PMC4734157

[158] Ma H Feng PH Yu SN Lu ZH Yu Q Chen J. Identification and validation of TNFRSF4 as a high-profile biomarker for prognosis and immunomodulation in endometrial carcinoma. BMC Cancer. (2022) 22:543. doi: 10.1186/s12885-022-09654-6, PMID: 35562682 PMC9107201

[159] Chang CY Lu YA Ting WC Shen TD Peng WC. An artificial immune system with bootstrap sampling for the diagnosis of recurrent endometrial cancers. Open Med (Wars). (2021) 16:237–45. doi: 10.1515/med-2021-0226, PMID: 33585700 PMC7863001

[160] Zhao Q Li Y Wang T. Development and validation of prediction model for early warning of ovarian metastasis risk of endometrial carcinoma. Med (Baltimore). (2023) 102:e35439. doi: 10.1097/MD.0000000000035439, PMID: 37832099 PMC10578755

[161] Zhao X Xu J Shui Y Xu M Hu J Liu X. PermuteDDS: a permutable feature fusion network for drug-drug synergy prediction. J Cheminform. (2024) 16:41. doi: 10.1186/s13321-024-00839-8, PMID: 38622663 PMC11017561

[162] Mater AC Coote ML. Deep learning in chemistry. J Chem Inf Model. (2019) 59:2545–59. doi: 10.1021/acs.jcim.9b00266, PMID: 31194543

[163] Holbeck SL Camalier R Crowell JA Govindharajulu JP Hollingshead M Anderson LW. The national cancer institute ALMANAC: A comprehensive screening resource for the detection of anticancer drug pairs with enhanced therapeutic activity. Cancer Res. (2017) 77:3564–76. doi: 10.1158/0008-5472.CAN-17-0489, PMID: 28446463 PMC5499996

[164] Preuer K Lewis RPI Hochreiter S Bender A Bulusu KC Klambauer G. DeepSynergy: predicting anti-cancer drug synergy with Deep Learning. Bioinformatics. (2018) 34:1538–46. doi: 10.1093/bioinformatics/btx806, PMID: 29253077 PMC5925774

[165] Castelvecchi D. Can we open the black box of AI? Nature. (2016) 538:20–3. doi: 10.1038/538020a, PMID: 27708329

[166] Peng Z Ding Y Zhang P Lv X Li Z Zhou X. Artificial intelligence application for anti-tumor drug synergy prediction. Curr Med Chem. (2024) 31:6572–85. doi: 10.2174/0109298673290777240301071513, PMID: 39420717

[167] Bongini P Scarselli F Bianchini M Dimitri GM Pancino N Liò P. Modular multi-source prediction of drug side-effects with DruGNN. (2023) 20:1211–20. doi: 10.48550/arXiv.2202.08147, PMID: 35576419

[168] Krasoulis A Antonopoulos N Pitsikalis V Theodorakis S. DENVIS: scalable and high-throughput virtual screening using graph neural networks with atomic and surface protein pocket features. J Chem Inf Model. (2022) 62:4642–59. doi: 10.1021/acs.jcim.2c01057, PMID: 36154119

[169] Nguyen T Le H Quinn TP Nguyen T Le TD Venkatesh S. GraphDTA: predicting drug-target binding affinity with graph neural networks. Bioinformatics. (2021) 37:1140–7. doi: 10.1093/bioinformatics/btaa921, PMID: 33119053

[170] Sun Z Huang S Jiang P Hu P. DTF: Deep Tensor Factorization for predicting anticancer drug synergy. Bioinformatics. (2020) 36:4483–9. doi: 10.1093/bioinformatics/btaa287, PMID: 32369563

[171] Liu Q Xie L. TranSynergy: Mechanism-driven interpretable deep neural network for the synergistic prediction and pathway deconvolution of drug combinations. PloS Comput Biol. (2021) 17:e1008653. doi: 10.1371/journal.pcbi.1008653, PMID: 33577560 PMC7906476

[172] Cifci D Foersch S Kather JN. Artificial intelligence to identify genetic alterations in conventional histopathology. J Pathol. (2022) 257:430–44. doi: 10.1002/path.5898, PMID: 35342954

[173] Noorbakhsh J Farahmand S Foroughi Pour A Namburi S Caruana D Rimm D. Deep learning-based cross-classifications reveal conserved spatial behaviors within tumor histological images. Nat Commun. (2020) 11:6367. doi: 10.1038/s41467-020-20030-5, PMID: 33311458 PMC7733499

[174] Miotto R Wang F Wang S Jiang X Dudley JT. Deep learning for healthcare: review, opportunities and challenges. Brief Bioinform. (2018) 19:1236–46. doi: 10.1093/bib/bbx044, PMID: 28481991 PMC6455466

[175] Wang S Yang DM Rong R Zhan X Fujimoto J Liu H. Artificial intelligence in lung cancer pathology image analysis. Cancers (Basel). (2019) 11:1673. doi: 10.3390/cancers11111673, PMID: 31661863 PMC6895901

[176] Foroughi Pour A White BS Park J Sheridan TB Chuang JH. Deep learning features encode interpretable morphologies within histological images. Sci Rep. (2022) 12:9428. doi: 10.1038/s41598-022-13541-2, PMID: 35676395 PMC9177767

[177] Sun H Zeng X Xu T Peng G Ma Y. Computer-aided diagnosis in histopathological images of the endometrium using a convolutional neural network and attention mechanisms. IEEE J BioMed Health Inform. (2020) 24:1664–76. doi: 10.1109/JBHI.2019.2944977, PMID: 31581102

[178] Farahani H Boschman J Farnell D Darbandsari A Zhang A Ahmadvand P. Deep learning-based histotype diagnosis of ovarian carcinoma whole-slide pathology images. Mod Pathol. (2022) 35:1983–90. doi: 10.1038/s41379-022-01146-z, PMID: 36065012

[179] Song J Im S Lee SH Jang HJ. Deep learning-based classification of uterine cervical and endometrial cancer subtypes from whole-slide histopathology images. Diagnostics (Basel). (2022) 12:2623. doi: 10.3390/diagnostics12112623, PMID: 36359467 PMC9689570

[180] Riasatian A Babaie M Maleki D Kalra S Valipour M Hemati S. Fine-Tuning and training of densenet for histopathology image representation using TCGA diagnostic slides. Med Image Anal. (2021) 70:102032. doi: 10.1016/j.media.2021.102032, PMID: 33773296

[181] Kumar-Sinha C Chinnaiyan AM. Precision oncology in the age of integrative genomics. Nat Biotechnol. (2018) 36:46–60. doi: 10.1038/nbt.4017, PMID: 29319699 PMC6364676

[182] Jumper J Evans R Pritzel A Green T Figurnov M Ronneberger O. Highly accurate protein structure prediction with AlphaFold. Nature. (2021) 596:583–9. doi: 10.1038/s41586-021-03819-2, PMID: 34265844 PMC8371605

[183] Yang Y Yang Y Yang J Zhao X Wei X. Tumor microenvironment in ovarian cancer: function and therapeutic strategy. Front Cell Dev Biol. (2020) 8:758. doi: 10.3389/fcell.2020.00758, PMID: 32850861 PMC7431690

[184] Zhang AW McPherson A Milne K Kroeger DR Hamilton PT Miranda A. Interfaces of Malignant and immunologic clonal dynamics in ovarian cancer. Cell. (2018) 173:1755–1769.e22. doi: 10.1016/j.cell.2018.03.073, PMID: 29754820

[185] Teyssonnière EM Trébulle P Muenzner J Loegler V Ludwig D Amari F. Species-wide quantitative transcriptomes and proteomes reveal distinct genetic control of gene expression variation in yeast. Proc Natl Acad Sci U.S.A. (2024) 121:e2319211121. doi: 10.1073/pnas.2319211121, PMID: 38696467 PMC11087752

[186] Teng PN Schaaf JP Abulez T Hood BL Wilson KN Litzi TJ. ProteoMixture: A cell type deconvolution tool for bulk tissue proteomic data. iScience. (2024) 27:109198. doi: 10.1016/j.isci.2024.109198, PMID: 38439970 PMC10910246

[187] Tan P Chen X Zhang H Wei Q Luo K. Artificial intelligence aids in development of nanomedicines for cancer management. Semin Cancer Biol. (2023) 89:61–75. doi: 10.1016/j.semcancer.2023.01.005, PMID: 36682438

[188] Akinci D’Antonoli T Cavallo AU Vernuccio F Stanzione A Klontzas ME Cannella R. Reproducibility of radiomics quality score: an intra- and inter-rater reliability study. Eur Radiol. (2024) 34:2791–804. doi: 10.1007/s00330-023-10217-x, PMID: 37733025 PMC10957586

[189] Shen D Wu G Suk HI. Deep learning in medical image analysis. Annu Rev BioMed Eng. (2017) 19:221–48. doi: 10.1146/annurev-bioeng-071516-044442, PMID: 28301734 PMC5479722

[190] Fortin JP Parker D Tunç B Watanabe T Elliott MA Ruparel K. Harmonization of multi-site diffusion tensor imaging data. Neuroimage. (2017) 161:149–70. doi: 10.1016/j.neuroimage.2017.08.047, PMID: 28826946 PMC5736019

[191] Cohen MD. Radiation risks of medical imaging. Radiology. (2013) 266:995. doi: 10.1148/radiol.12122215, PMID: 23431231

[192] Esteva A Kuprel B Novoa RA Ko J Swetter SM Blau HM. Dermatologist-level classification of skin cancer with deep neural networks. Nature. (2017) 542:115–8. doi: 10.1038/nature21056, PMID: 28117445 PMC8382232

[193] Gulshan V Peng L Coram M Stumpe MC Wu D Narayanaswamy A. Development and validation of a deep learning algorithm for detection of diabetic retinopathy in retinal fundus photographs. JAMA. (2016) 316:2402–10. doi: 10.1001/jama.2016.17216, PMID: 27898976

[194] Ciompi F Chung K van Riel SJ Setio AAA Gerke PK Jacobs C. Towards automatic pulmonary nodule management in lung cancer screening with deep learning. Sci Rep. (2017) 7:46479. doi: 10.1038/srep46479, PMID: 28422152 PMC5395959

[195] Menze BH Jakab A Bauer S Kalpathy-Cramer J Farahani K Kirby J. The multimodal brain tumor image segmentation benchmark (BRATS). IEEE Trans Med Imaging. (2015) 34:1993–2024. doi: 10.1109/TMI.2014.2377694, PMID: 25494501 PMC4833122

[196] Liu S Liu S Cai W Che H Pujol S Kikinis R. Multimodal neuroimaging feature learning for multiclass diagnosis of Alzheimer’s disease. IEEE Trans BioMed Eng. (2015) 62:1132–40. doi: 10.1109/TBME.2014.2372011, PMID: 25423647 PMC4394860

[197] Eiber M Weirich G Holzapfel K Souvatzoglou M Haller B Rauscher I. Simultaneous ^68^Ga-PSMA HBED-CC PET/MRI improves the localization of primary prostate cancer. Eur Urol. (2016) 70:829–36. doi: 10.1016/j.eururo.2015.12.053, PMID: 26795686

[198] Zhou M Scott J Chaudhury B Hall L Goldgof D Yeom KW. Radiomics in brain tumor: image assessment, quantitative feature descriptors, and machine-learning approaches. AJNR Am J Neuroradiol. (2018) 39:208–16. doi: 10.3174/ajnr.A5391, PMID: 28982791 PMC5812810

[199] Vallières M Freeman CR Skamene SR El Naqa I. A radiomics model from joint FDG-PET and MRI texture features for the prediction of lung metastases in soft-tissue sarcomas of the extremities. Phys Med Biol. (2015) 60:5471–96. doi: 10.1088/0031-9155/60/14/5471, PMID: 26119045

[200] Vallières M Kay-Rivest E Perrin LJ Liem X Furstoss C Aerts HJWL. Radiomics strategies for risk assessment of tumour failure in head-and-neck cancer. Sci Rep. (2017) 7:10117. doi: 10.1038/s41598-017-10371-5, PMID: 28860628 PMC5579274

[201] Men K Dai J Li Y. Automatic segmentation of the clinical target volume and organs at risk in the planning CT for rectal cancer using deep dilated convolutional neural networks. Med Phys. (2017) 44:6377–89. doi: 10.1002/mp.12602, PMID: 28963779

[202] Jha S Topol EJ. Adapting to artificial intelligence: radiologists and pathologists as information specialists. JAMA. (2016) 316:2353–4. doi: 10.1001/jama.2016.17438, PMID: 27898975

[203] Howard FM Dolezal J Kochanny S Schulte J Chen H Heij L. The impact of site-specific digital histology signatures on deep learning model accuracy and bias. Nat Commun. (2021) 12:4423. doi: 10.1038/s41467-021-24698-1, PMID: 34285218 PMC8292530

[204] Schnellinger EM Yang W Kimmel SE. Comparison of dynamic updating strategies for clinical prediction models. Diagn Progn Res. (2021) 5:20. doi: 10.1186/s41512-021-00110-w, PMID: 34865652 PMC8647501

[205] Eche T Schwartz LH Mokrane FZ Dercle L. Toward generalizability in the deployment of artificial intelligence in radiology: role of computation stress testing to overcome underspecification. Radiol Artif Intell. (2021) 3:e210097. doi: 10.1148/ryai.2021210097, PMID: 34870222 PMC8637230

[206] Saldanha OL Quirke P West NP James JA Loughrey MB Grabsch HI. Swarm learning for decentralized artificial intelligence in cancer histopathology. Nat Med. (2022) 28:1232–9. doi: 10.1038/s41591-022-01768-5, PMID: 35469069 PMC9205774

[207] Ding K Zhou M Wang H Gevaert O Metaxas D Zhang S. A large-scale synthetic pathological dataset for deep learning-enabled segmentation of breast cancer. Sci Data. (2023) 10:231. doi: 10.1038/s41597-023-02125-y, PMID: 37085533 PMC10121551

[208] Lu MY Williamson DFK Chen TY Chen RJ Barbieri M Mahmood F. Data-efficient and weakly supervised computational pathology on whole-slide images. Nat BioMed Eng. (2021) 5:555–70. doi: 10.1038/s41551-020-00682-w, PMID: 33649564 PMC8711640

[209] Rubin DL. Artificial intelligence in imaging: the radiologist’s role. J Am Coll Radiol. (2019) 16:1309–17. doi: 10.1016/j.jacr.2019.05.036, PMID: 31492409 PMC6733578

[210] Savadjiev P Chong J Dohan A Vakalopoulou M Reinhold C Paragios N. Demystification of AI-driven medical image interpretation: past, present and future. Eur Radiol. (2019) 29:1616–24. doi: 10.1007/s00330-018-5674-x, PMID: 30105410

[211] Price WN 2nd Gerke S Cohen IG. How much can potential jurors tell us about liability for medical artificial intelligence? J Nucl Med. (2021) 62:15–6. doi: 10.2967/jnumed.120.257196, PMID: 33158905

[212] Coppola F Faggioni L Gabelloni M De Vietro F Mendola V Cattabriga A. Human, all too human? An all-around appraisal of the “Artificial intelligence revolution” in medical imaging. Front Psychol. (2021) 12:710982. doi: 10.3389/fpsyg.2021.710982, PMID: 34650476 PMC8505993

[213] Triberti S Durosini I Pravettoni G. A “Third wheel” Effect in health decision making involving artificial entities: A psychological perspective. Front Public Health. (2020) 8:117. doi: 10.3389/fpubh.2020.00117, PMID: 32411641 PMC7199477

[214] Coppola F Faggioni L Regge D Giovagnoni A Golfieri R Bibbolino C. Artificial intelligence: radiologists’ expectations and opinions gleaned from a nationwide online survey. Radiol Med. (2021) 126:63–71. doi: 10.1007/s11547-020-01205-y, PMID: 32350797

[215] Karako K Chen Y Tang W. On medical application of neural networks trained with various types of data. Biosci Trends. (2019) 12:553–9. doi: 10.5582/bst.2018.01264, PMID: 30555113

[216] Choi E Bahadori MT Schuetz A Stewart WF Sun J. RETAIN: interpretable predictive model in healthcare using reverse time attention mechanism. (2016), 3512–20. doi: 10.48550/arXiv.1608.05745

[217] Huang S Yang J Shen N Xu Q Zhao Q. Artificial intelligence in lung cancer diagnosis and prognosis: Current application and future perspective. Semin Cancer Biol. (2023) 89:30–7. doi: 10.1016/j.semcancer.2023.01.006, PMID: 36682439

